# Supplementary material for: Single-cell lineage tracing by integrating CRISPR-Cas9 mutations with transcriptomic data
Source: Nat Commun. 2020 Jun 16;11:3055. doi: 10.1038/s41467-020-16821-5 (PMC7298005; doi:10.1038/s41467-020-16821-5)
Supplement: Supplementary file 1 — Supplementary Information [file 41467_2020_16821_MOESM1_ESM.pdf]

Supplementary Information for Single-cell Lineage Tracing by Integrating CRISPR-Cas9 Mutations with Transcriptomic Data

Hamim Zafar[1,2,3,†], Chieh Lin[4,†], Ziv Bar-Joseph[3,4,*]
[1]Department of Computer Science and Engineering
[2]Department of Biological Sciences and Bioengineering
Indian Institute of Technology Kanpur
[3]Computational Biology Department, [4]Machine Learning Department
School of Computer Science, Carnegie Mellon University
† *These authors contributed equally*
* *Corresponding author, zivbj@cs.cmu.edu*

June 5, 2020

Contents

1 Supplementary Methods **2**
1.1 Imputation of gene expression data . . . 2
1.2 Normalization of scRNA-seq data . . . 2
1.3 Prior distribution for computing expression likelihood . . . 2
1.4 Proposal for cluster matching . . . 3
1.5 Visualizing LinTIMaT trees . . . 4

2 Supplementary Discussion **5**
2.1 Testing LinTIMaT using a benchmark Caenorhabditis elegans dataset . . . 5
2.2 Recovering convergent and divergent differentiation using LinTIMaT . . . 6
2.3 Divergent lineage trajectories inferred by LinTIMaT for scGESTALT datasets . . . 8
2.4 Applying LinTIMaT on ScarTrace zebrafish dataset . . . 8
2.5 Computing ARI for cell clustering based on Maximum Parsimony lineage trees . . . 9
2.6 ARI values for lineages reconstructed by LinTIMaT . . . 9
2.7 LinTIMaT runtime . . . 9

3 Supplementary Figures **10**

4 Supplementary Tables **32**

1 Supplementary Methods

1.1 Imputation of gene expression data

The scRNA-seq data in both scGESTALT and ScarTrace datasets displayed a high dropout rate (about 94% entries were 0). To address this, we decided to impute the scRNA-seq data. To ensure that LinTIMaT's likelihood function is not sensitive to the imputation method, we tested two different imputation methods named DrImpute [1] and SAVER [2]. We observed that LinTIMaT's results were not dependent on the imputation method used and the cell clustering performance from the same lineage tree were similar for the different imputation methods (Supplementary Figure 21). We finally selected DrImpute as it has been shown to perform better in other experiments [3].

1.2 Normalization of scRNA-seq data

After imputing the scRNA-seq data using DrImpute, we normalized the data. For normalization, we divided each expression value by the sum of expression values for each cell and multiplied the expression value with a scaling factor 10000, which is a common scaling factor for UMI (unique molecular identifier) data.

1.3 Prior distribution for computing expression likelihood

LinTIMaT's expression likelihood function computes the probability of the expression data under a node based on two alternative hypotheses. The first hypothesis computes the marginal probability of the data being generated from a single cluster. For computing this marginal probability using Equation (7), we choose a univariate Gaussian distribution and the Normal-inverse-chi-squared (NIX) prior for β. NIX prior is the univariate version of normal-inverse-Wishart (NIW) prior, which is the prior suggested by BHC. We adopt the univariate version to reduce time and space complexity of LinTIMaT. NIX prior has the following parameters $\beta = (\mu_0, \kappa_0, \sigma_0^2, \nu_0)$, where μ_0 and σ_0^2 are the priors on the mean and variance of the Gaussian distribution. κ_0 and ν_0 are the confidence on the prior of the mean and variance respectively. The posterior parameters $\{\mu_v, \sigma_v^2, \kappa_v, \nu_v\}$ and the marginal probability for the subtree $\mathscr{T}^v$ rooted at node v under the NIX prior are derived

according to [4] and shown below

$$\kappa_v = \kappa_0 + n_v \tag{1}$$

$$\nu_v = \nu_0 + n_v \tag{2}$$

$$\mu_v = \frac{\kappa_0 \mu_0 + n_v \bar{\mathbf{y}}_v}{\kappa_v} \tag{3}$$

$$\sigma_v^2 = \frac{1}{\nu_v} \left(\nu_0 \sigma_0^2 + \sum_{\mathbf{y}^{(i)} \in \mathcal{Y}_g^v} (\mathbf{y}^{(i)} - \bar{\mathbf{y}}_v)^2 + \frac{n_v \kappa_0}{\kappa_v} (\mu_0 - \bar{\mathbf{y}}_v)^2 \right), \tag{4}$$

where $\bar{\mathbf{y}}_v$ is the sample mean of $\mathcal{Y}_g^v$, the gene expression values for gene g under the node v. The marginal likelihood is given by

$$P(\mathcal{Y}_g^v | \mathscr{H}_1^v) = \frac{\Gamma(\nu_v/2)}{\Gamma(\nu_0/2)} \sqrt{\frac{\kappa_0}{\kappa_v}} \frac{(\nu_0 \sigma_0^2)^{\nu_0/2}}{(\nu_v \sigma_k^2)^{\nu_v/2}} \frac{1}{\pi^{n_v/2}} \tag{5}$$

For the hyperparameters of β, we set μ_0 and σ_0^2 to sample mean and sample variance based on all single cells, $\mu_0 = \frac{1}{N} \sum_{c=1}^{N} \mathcal{Y}_{cg}, \sigma_0^2 = \sum_{c=1}^{N} (\mathcal{Y}_{cg} - \mu_0)^2$. The confidence parameters are set to 1, $\kappa_0 = \nu_0 = 1$.

1.4 Proposal for cluster matching

We use a two-step heuristic search algorithm for inferring the invariant lineage and the corresponding cluster matching. We employ two different proposals for proposing a new cluster matching for the two steps of the invariant lineage search algorithm respectively.

1. Let us assume $\mathcal{M}_{old}$ denotes the current matching from which we want to propose a new cluster matching. In $\mathcal{M}_{old}$, K clusters (leaves) in the invariant lineage are matched to K clusters in each individual lineage. Let $\{c_1, \ldots, c_K\}$ denotes the K clusters in the invariant lineage. In $\mathcal{M}_{old}$, for individual lineage $\mathscr{T}_i^c$ ($i \in \{1, \ldots, I\}$), let $l_i^{c_x}$ and $l_i^{c_y}$ denote the clusters that match with clusters c_x and c_y in the invariant respectively. We can propose a new cluster matching $\mathcal{M}_{new}^i$ by swapping the matchings of $l_i^{c_x}$ and $l_i^{c_y}$ with c_x and c_y. There are $\mathcal{O}(K^2)$ such possible swaps. For each such swap η, we compute a score $\Sigma_\eta = \Delta_{\mathcal{S}}^\eta + \Delta_{\mathcal{E}}^\eta$, where $\Delta_{\mathcal{S}}^\eta$ denotes the improvement in $\mathcal{D}_{\mathcal{S}}$ after the swap and $\Delta_{\mathcal{E}}^\eta$ denotes the improvement in $\mathcal{D}_{\mathcal{E}}$ after the swap. The swaps for which both $\Delta_{\mathcal{E}}^\eta$ and Σ_η are positive are considered to be good swaps.

One such good swap is chosen randomly to propose a new matching $\mathcal{M}^i_{new}$. If no good swap is available, no swapping is performed. This is done sequentially for all I individual lineages to produce a new cluster matching $\mathcal{M}_{new}$.

2. In the second step of the search algorithm, we perform a random swap to propose a new matching. For invariant lineage, we randomly choose two clusters c_x and c_y, and in the individual lineage $\mathscr{T}_i^c$, we swap their matchings with $l_i^{c_x}$ and $l_i^{c_y}$.

1.5 Visualizing LinTIMaT trees

Following [5], individual cells (leaves) in the lineage trees were annotated by their corresponding cell types. LinTIMaT lineage trees were converted into JSON objects with annotated cell type membership using custom python scripts. Finally, the JSON objects were visualized using the modified custom scripts of [5] using D3 software framework. The visualization web page also displays additional information on each tree node such as mutations and cell type proportions.

2 Supplementary Discussion

2.1 Testing LinTIMaT using a benchmark Caenorhabditis elegans dataset

For simulated datasets with fixed mutation rate per cell division, for all values of mutation rates, LinTIMaT achieved higher accuracy in lineage reconstruction compared to that of MP and NJ. For lower mutation rates ($\mu \leq 0.15$), LinTIMaT achieved upto 41.64% improvement in mean lineage reconstruction accuracy over that of MP (41.64%, 14.44% and 32.02% improvement for $\mu = 0.05$, $\mu = 0.1$ and $\mu = 0.15$ respectively) and upto 29.45% improvement over that of NJ (29.45%, 15.19% and 21.81% improvement for $\mu = 0.05$, $\mu = 0.1$ and $\mu = 0.15$ respectively). For these values of mutation rates, LinTIMaT also achieved lower RF distance compared to the FP and FN distances for the trees inferred by MP and NJ (Supplementary Figure 2). This indicates that by utilizing the transcriptomic data, LinTIMaT was indeed able to recover some of the branchings of the reference lineage that did not harbor any CRISPR mutations (which were indeed not recovered by MP or NJ). Performance of MP and NJ improved with an increase in mutation rate but even for datasets with higher mutation rates ($\mu \geq 0.2$), LinTIMaT was able to achieve better solution compared to that of MP (12.9%, 9.3% and 11.89% improvement in mean lineage reconstruction accuracy for $\mu = 0.2$, $\mu = 0.25$ and $\mu = 0.3$ respectively) and NJ (16.57%, 9.7% and 9% improvement in mean lineage reconstruction accuracy for $\mu = 0.2$, $\mu = 0.25$ and $\mu = 0.3$ respectively). Next, we simulated datasets for which the mutation rate differed between sites. In such cases, sites with higher mutation rate could saturate early in contrast to sites with lower mutation rate that might not harbor any mutation at all. For such datasets, LinTIMaT achieved higher accuracy (13.56% – 30.37% improvement) and lower RF distance compared to that of MP and NJ (Supplementary Figure 3). This indicates that LinTIMaT's performance is more robust to the increase in complexity in the CRISPR mutational history. CRISPR activity affecting multiple targets simultaneously can result in erasing some of the earlier lineage records [6]. Such dropouts of CRISPR mutations have been shown to have significant impact on the lineage reconstruction accuracy [7]. In order to assess the performance of LinTIMaT in the presence of mutation dropouts, we simulated datasets with different dropout rates for a fixed mutation rate ($\epsilon_d = \{1, 2, 3\}$, $\mu = 0.15$) where ϵ_d denotes the expected number of dropout events in the cell lineage. As expected, lineage reconstruction accuracy of all methods decreased as the number of dropouts increased. However, for all settings, LinTIMaT

achieved better accuracy than MP (17.45%, 49.53% and 33.53% better mean accuracy for $\epsilon_d = 1$, $\epsilon_d = 2$ and $\epsilon_d = 3$ respectively) as well as NJ (8.8%, 31.37% and 28.3% better mean accuracy for $\epsilon_d = 1$, $\epsilon_d = 2$ and $\epsilon_d = 3$ respectively) indicating that in the presence of dropouts, LinTIMaT is able to recover more accurate branchings in the cell lineage compared to that of MP and NJ. This is further indicated by LinTIMaT's smaller RF distance for all settings compared to that of MP and NJ (Supplementary Figure 4).

2.2 Recovering convergent and divergent differentiation using LinTIMaT

To evaluate LinTIMaT's ability in recovering convergent differentiation where cells converge to similar transcriptional state despite having distinct origin, we selected forebrain neuron cells from hypothalamus (cell type 27 in ZF1 dataset of scGESTALT) and divided into two groups (G1 and G2 containing 11 and 10 cells respectively). We simulated lineages on 100 cells and these two groups of cells were placed in two different subtrees. The other 79 cells were chosen from different neuron (forebrain, midbrain and hindbrain), progenitor, blood and mixed cell types. We simulated lineages under two different settings regarding the common ancestor of the cells. In the first setting, the root of the lineage tree is the common ancestor of the two groups and they did not share any CRISPR mutation (see Supplementary Figure 6a for an example). In the second setting, the two groups diverged very early on (child of root node being their most recent common ancestor) and possibly shared some CRISPR mutation (see Supplementary Figure 7a for an example). For each setting, we simulated CRISPR mutations on the simulated lineages under three experimental conditions ($e1 : \mu = 0.15,$ dropout in one subtree; $e2 : \mu = 0.15,$ dropouts in both subtrees; and $e3 : \mu = 0.1,$ dropouts in both subtrees) with increasing difficulties and tested whether LinTIMaT was able to infer the lineage relationship between these two groups despite them being transcriptionally similar. The lineage reconstruction error was measured by pairwise leaf shortest-path distance [8, 9] between the two groups. For all experimental conditions, LinTIMaT's lineage reconstruction error was lower compared to the average lineage reconstruction error resulting from placing the two groups in the same subtree (Supplementary Figures 6b and 7b). For both experimental settings, LinTIMaT was able to correctly place the two groups in different subtrees (5 (out of 5) times for $e1$ and $e2$, 4 (out of 5) times for $e3$) (see Supplementary Figures 6c and 7c). For the example where LinTIMaT incorrectly placed the groups in the same subtree, on examining the mutational

data, we observed that the two groups were separated by only two ancestral branches (the groups were close to their MRCA in the lineage) and both of these branches were affected by mutation dropout. As a result, there was no mutational information separating the two groups, in fact, they shared a common mutation. This example presents a convergent differentiation scenario where the use of expression data can lead to less accurate reconstruction due to the absence of any mutation information. However, any lineage reconstruction method relying on the mutation information (such as MP or NJ) would have faced difficulty in this scenario due to the lack of mutation data. Also note that, results for this simulation experiment are based on a default setting for the weight parameter for the expression data. A very low weight for the expression data (compared to the weight for mutation data) may lead to different results that may not accurately reconstruct the lineage.

For assessing LinTIMaT's ability to correctly infer the lineage relationship between two groups of cells that shared a close ancestry but diverged into distinct transcriptional states (different cell types), we selected two groups of cells, the first group (G1) consisting of 11 forebrain neuron cells from hypothalamus (cell type 27 in ZF1 dataset of scGESTALT) and the second group (G2) consisting of 10 progenitor cells (cell type 8 in ZF1 dataset of scGESTALT). We simulated lineages on 100 cells and these two groups of cells were placed next to each other (see Supplementary Figure 8a for an example). The other 79 cells were chosen from different neuron (forebrain, midbrain and hindbrain), progenitor, blood and mixed cell types. CRISPR mutations were simulated on the cell lineages under two experimental conditions ($e1 : \mu = 0.15, 1$ dropout in the subtree; $e2 : \mu = 0.1, 1$ dropout in the subtree) and we tested whether LinTIMaT was able to place them together in the same subtree despite them being from different cell types. The dropout was introduced on the path from the root to the common ancestor (MRCA) of the two groups. For different experimental conditions, LinTIMaT achieved lower lineage reconstruction error compared to a randomized error that represents an average lineage reconstruction error resulting from placing the two groups in different subtrees (Supplementary Figure 8b) and correctly placed them in the same subtree (5 (out of 5) times for $e1$ and $e2$, Supplementary Figure 8c).

2.3 Divergent lineage trajectories inferred by LinTIMaT for scGESTALT datasets

The lineage trees reconstructed by LinTIMaT for scGESTALT datasets revealed divergent lineage trajectories. For example, for ZF3, LinTIMaT lineage tree displayed three major subtrees under clade a (a1, a2 and a3 respectively), with a1 being sub-divided into three major clusters. Clade a1 had three major clusters consisting mostly of progenitor cells, hindbrain and forebrain neurons respectively. The constructed tree indicates that the $her4.1^+$ and $atoh1c^+$ progenitor cells [10, 11] are closely related to $pax6b^+$ granule cells [12] in hindbrain, $gad2^+$ neurons in ventral forebrain [13], and $fezf1^+$ neurons [14] in hypothalamus region. On the other hand, $pitx2^+$ and $prdx1^+$ neurons [15] in forebrain (clade a2) were determined to be related to radial glia cells (clade a3). These results demonstrate LinTIMaT's ability to elucidate complex lineage relationships of cells.

2.4 Applying LinTIMaT on ScarTrace zebrafish dataset

ScarTrace [16] is a lineage tracing experimental method that combined CRISPR-Cas9 lineage recording with SORT-seq transcriptome profiling for detecting mutational scars and scRNA-seq from single cells. In the original study, ScarTrace was applied on different organs of zebrafish (adult brain, eyes, caudal fin, etc.) for different replicates. We selected 2 zebrafish replicates (R2 and R3) for which the adult brain and eyes were profiled. After preprocessing (selecting the cells that have both mutation and expression data), R2 and R3 consisted of 1320 and 749 cells respectively with around 16K genes and 60-80 unique mutational scars. To match the cell type distribution of the cells in both replicates, we further selected 750 cells (out of 1320) for R2 for applying LinTIMaT. The large lineage trees inferred by LinTIMaT for R2 and R3 can be visualized at `https://jessica1338.github.io/LinTIMaT/`. For comparison, we also reconstructed lineage trees using MP for these two fishes. LinTIMaT was able to separate cells based on their cell types that were all clustered together by MP due to their shared mutational barcode. Clade a in R2 lineage tree (Supplementary Figure 14) is one such example, where LinTIMaT was able to separate left midbrain neurons, rod cells and immune cells into two subtrees in contrast to MP that clustered all these cells together. Similar examples (Supplementary Figure 15) can also be seen in R3 lineage tree, where in MP lineage, right eye neurons, immune cells and cells with unknown cell types were clustered together but LinTIMaT successfully assigned them into different subtrees under the same

mutational node. In addition, LinTIMaT lineage for R3 also displayed example (Supplementary Figure 16) where cells belonging to similar cell types but carrying different mutational barcodes were identified as a cluster. In clade c, LinTIMaT identified right midbrain neurons as a cluster that were otherwise in different branches and mixed with neurons from left midbrain and immune cells.

2.5 Computing ARI for cell clustering based on Maximum Parsimony lineage trees

To compare LinTIMaT lineages against Maximum Parsimony (MP) lineages from [5] for ZF1 and ZF3, we compared the cell clustering performance for the lineage trees. The cell clustering performance was measured by computing ARI. While LinTIMaT trees allow for inferring cell clusters based on gene expression data, MP lineage trees do not provide such option. For MP lineage trees, the unique barcodes can be treated as cell clusters. However, to be more thorough, we also cut the MP trees at different levels to obtain different possible cell clusterings. The ARI values for the clusterings obtained by cutting the MP trees at level 1-6 and the barcode level for both ZF1 and ZF3 are shown in Supplementary Table 1. For both fishes, the barcode level clustering achieved the highest ARI values. Consequently, these values were used for comparing MP trees against LinTIMaT trees.

2.6 ARI values for lineages reconstructed by LinTIMaT

We computed Adjusted Rand Index (ARI) for the cell clustering inferred from lineages reconstructed using LinTIMaT. ARI was 0.084 for ZF1 and 0.076 for ZF3 respectively.

2.7 LinTIMaT runtime

For measuring the runtime of LinTIMaT, we ran it on a CPU with 8 cores. When reconstructing the individual lineage for scGESTALT dataset (ZF3), runtime of LinTIMaT was 8.18 hours. For ScarTrace datasets (R3), LinTIMaT inferred the individual lineage in 14.08 hours. The runtime for reconstruction of the invariant lineage was 23.5 minutes for scGESTALT datasets and 88 minutes for ScarTrace datasets respectively.

3 Supplementary Figures

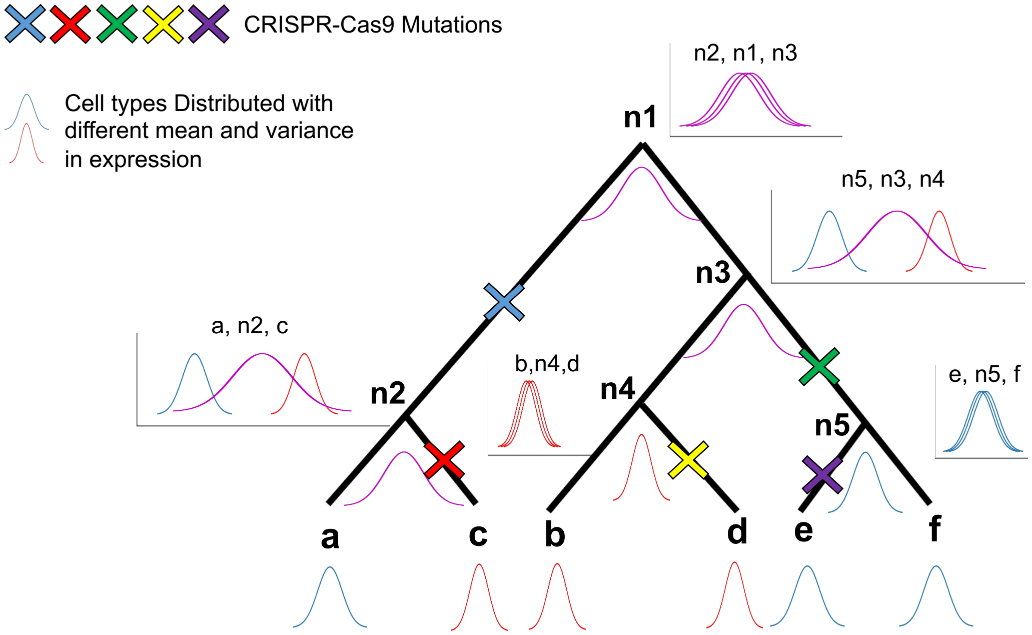

Supplementary Figure 1: Generative process of LinTIMaT. Different CRISPR-Cas9 mutations are acquired on the branches of the lineage. a,b,c,d,e,f represent different barcodes. a, e and f contain cells from cell type 1 (blue); b, c and d contain cells from cell type 2 (red). The gene-expression at an internal node follows a Gaussian distribution based on the cells in the subtree rooted at the node. If children have similar distribution, then the internal node will also have similar distribution (e.g., n1, n4, n5). If children have different distribution, the internal node will have a distribution with larger variance (e.g., n2, n3). Cells with similar expression can occur in distant branches of the cell lineage. For example, c has similar expression profile as b and d; a has similar expression profile as e and f but because of their different mutation profile, LinTIMaT is able place them on distant branches.

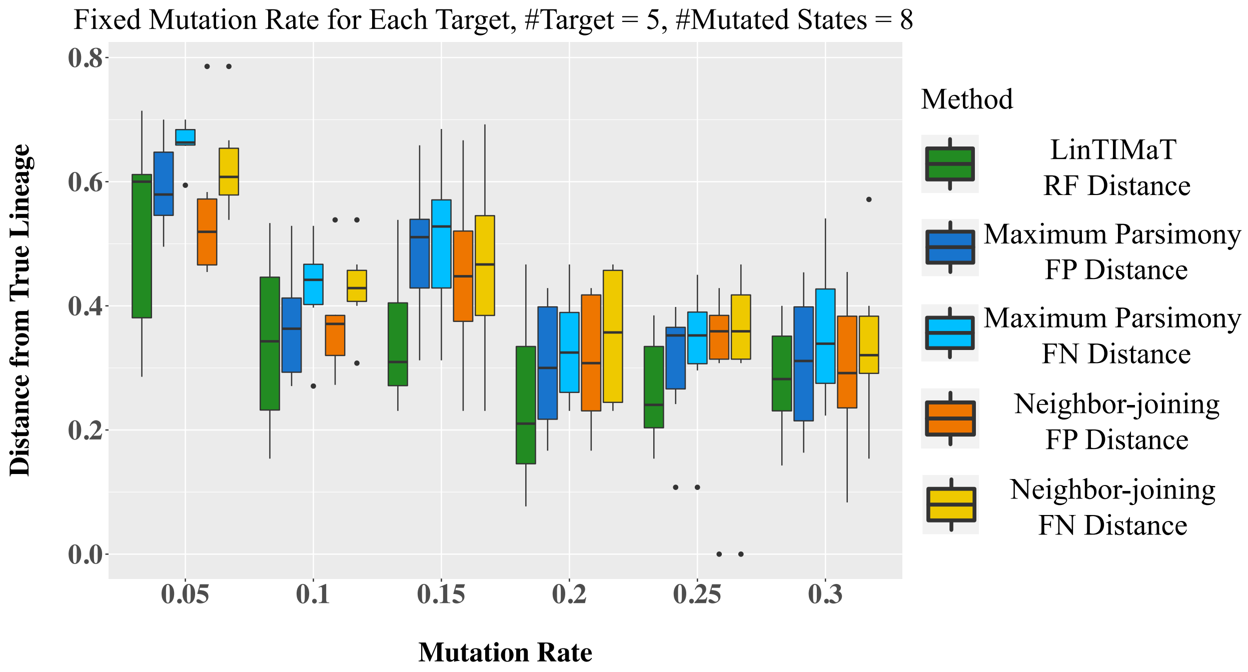

Supplementary Figure 2: Comparison of lineage reconstruction performance by LinTIMaT, Camin-Sokal Maximum Parsimony and Neighbor-joining with a lineage recorder of 5 CRISPR targets based on 16 cell *C. elegans* lineage over a range of mutation rates. The number of possible mutational states was set to 8. Fixed mutation rate was used for each CRISPR target. As a measure of performance, RF distance between the true and inferred lineage was computed for LinTIMaT, FP and FN distances between the true and inferred lineages were computed for Camin-Sokal Maximum Parsimony and Neighbor-joining. Lower distance corresponds to better lineage reconstruction. Each box-and-whisker plot summarizes results for 6 replicates with varying simulated CRISPR mutation data and experimental scRNA-seq data, where the box shows the interquartile range (IQR, the range between the 25th and 75th percentile) with the median value, whiskers indicate the maximum and minimum value within 1.5 times the IQR, also shown are outliers as black dots.

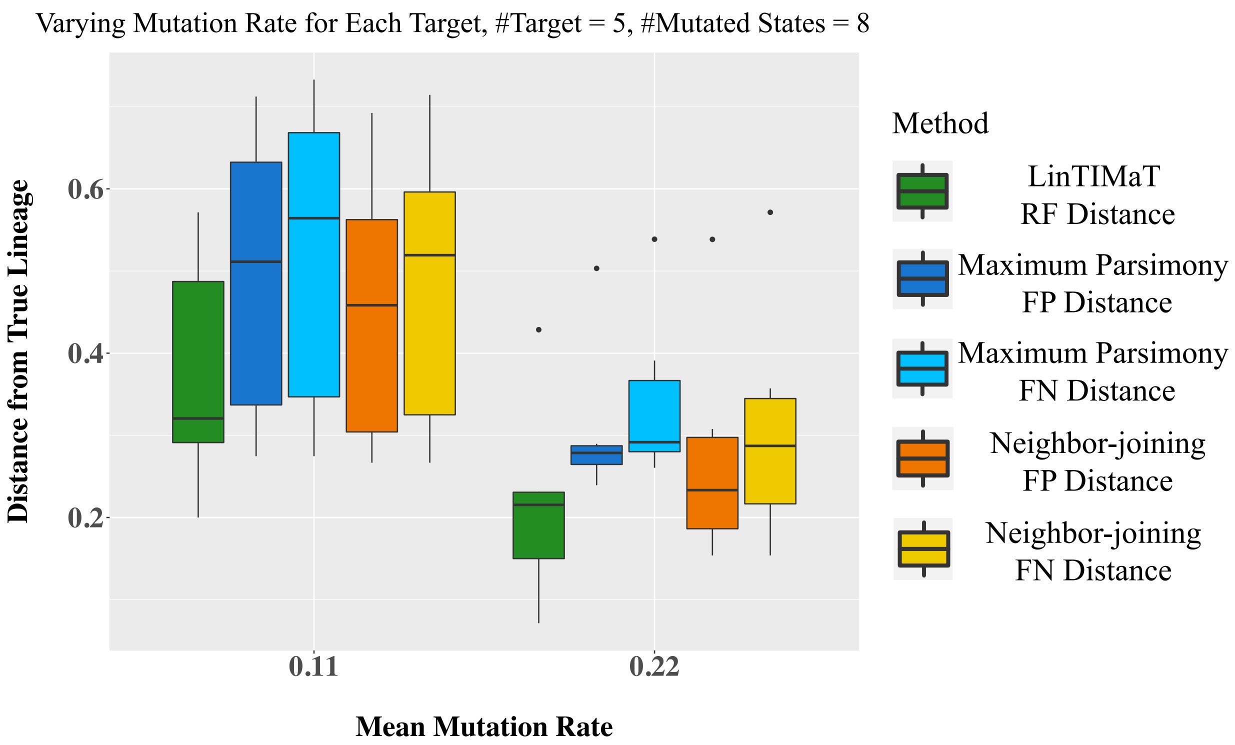

Supplementary Figure 3: Comparison of lineage reconstruction performance by LinTIMaT, Camin-Sokal Maximum Parsimony and Neighbor-joining based on 16 cell *C. elegans* lineage when mutation rate was varied from one target to another. As a measure of performance, RF distance between the true and inferred lineage was computed for LinTIMaT, FP and FN distances between the true and inferred lineages were computed for Camin-Sokal Maximum Parsimony and Neighbor-joining. Lower distance corresponds to better lineage reconstruction. Each box-and-whisker plot summarizes results for 6 replicates with varying simulated CRISPR mutation data and experimental scRNA-seq data, where the box shows the interquartile range (IQR, the range between the 25th and 75th percentile) with the median value, whiskers indicate the maximum and minimum value within 1.5 times the IQR, also shown are outliers as black dots.

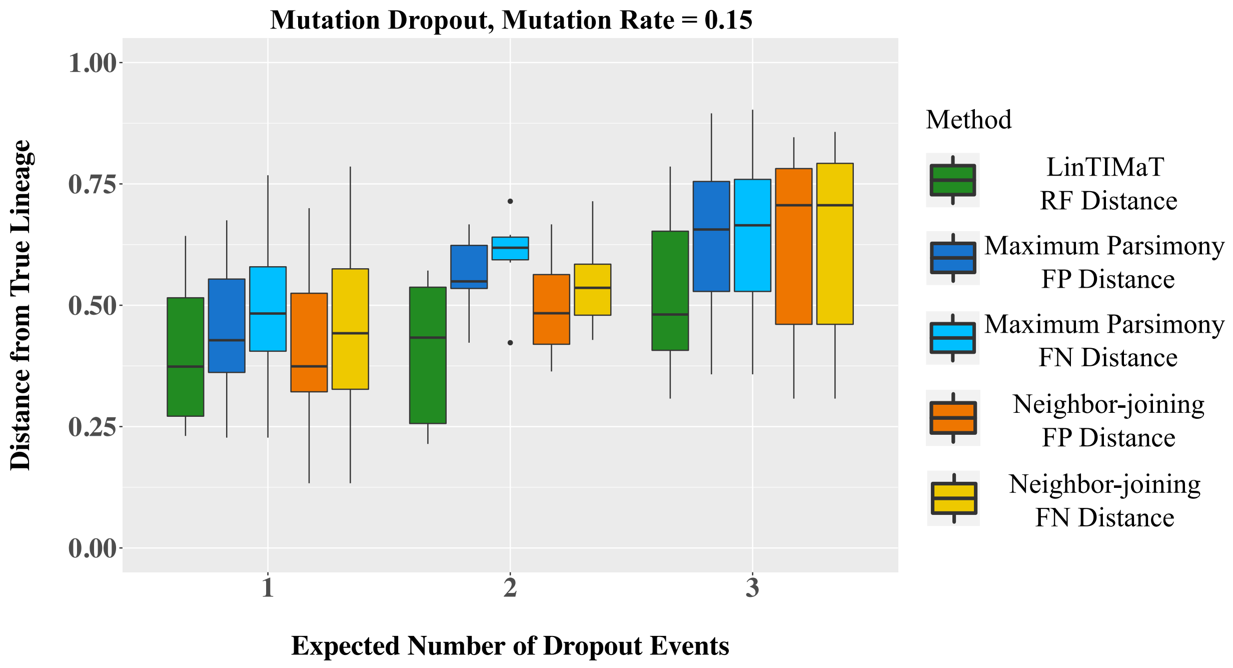

Supplementary Figure 4: Comparison of lineage reconstruction performance by LinTIMaT, Camin-Sokal Maximum Parsimony and Neighbor-joining based on 16 cell *C. elegans* lineage in the presence of mutation dropout. Fixed mutation rate, $\mu = 0.15$ was used for each CRISPR target. As a measure of performance, RF distance between the true and inferred lineage was computed for LinTIMaT, FP and FN distances between the true and inferred lineages were computed for Camin-Sokal Maximum Parsimony and Neighbor-joining. Lower distance corresponds to better lineage reconstruction. Each box-and-whisker plot summarizes results for 6 replicates with varying simulated CRISPR mutation data and experimental scRNA-seq data, where the box shows the interquartile range (IQR, the range between the 25th and 75th percentile) with the median value, whiskers indicate the maximum and minimum value within 1.5 times the IQR, also shown are outliers as black dots.

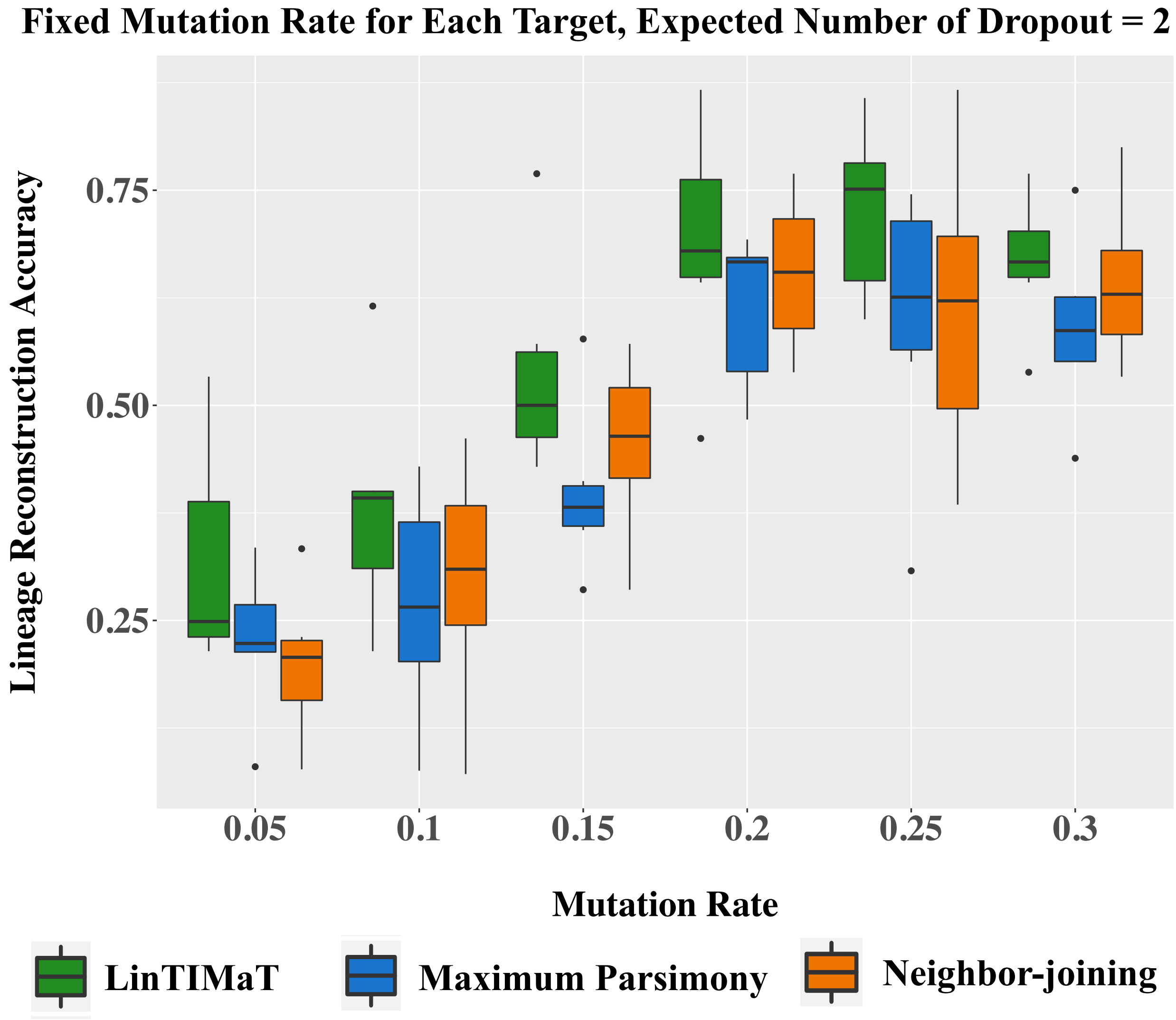

Supplementary Figure 5: Comparison of lineage reconstruction performance by LinTIMaT, Camin-Sokal Maximum Parsimony and Neighbor-joining based on 16 cell *C. elegans* lineage in the presence of mutation dropout. Fixed mutation rate was used for each CRISPR target. For each setting, 2 dropouts were introduced. Mutation rate was varied from $\mu = 0.05$ to $\mu = 0.3$. As a measure of performance, RF distance between the true and inferred lineage was computed for LinTIMaT, FP and FN distances between the true and inferred lineages were computed for Camin-Sokal Maximum Parsimony and Neighbor-joining. Lower distance corresponds to better lineage reconstruction. Each box-and-whisker plot summarizes results for 6 replicates with varying simulated CRISPR mutation data and experimental scRNA-seq data, where the box shows the interquartile range (IQR, the range between the 25th and 75th percentile) with the median value, whiskers indicate the maximum and minimum value within 1.5 times the IQR, also shown are outliers as black dots.

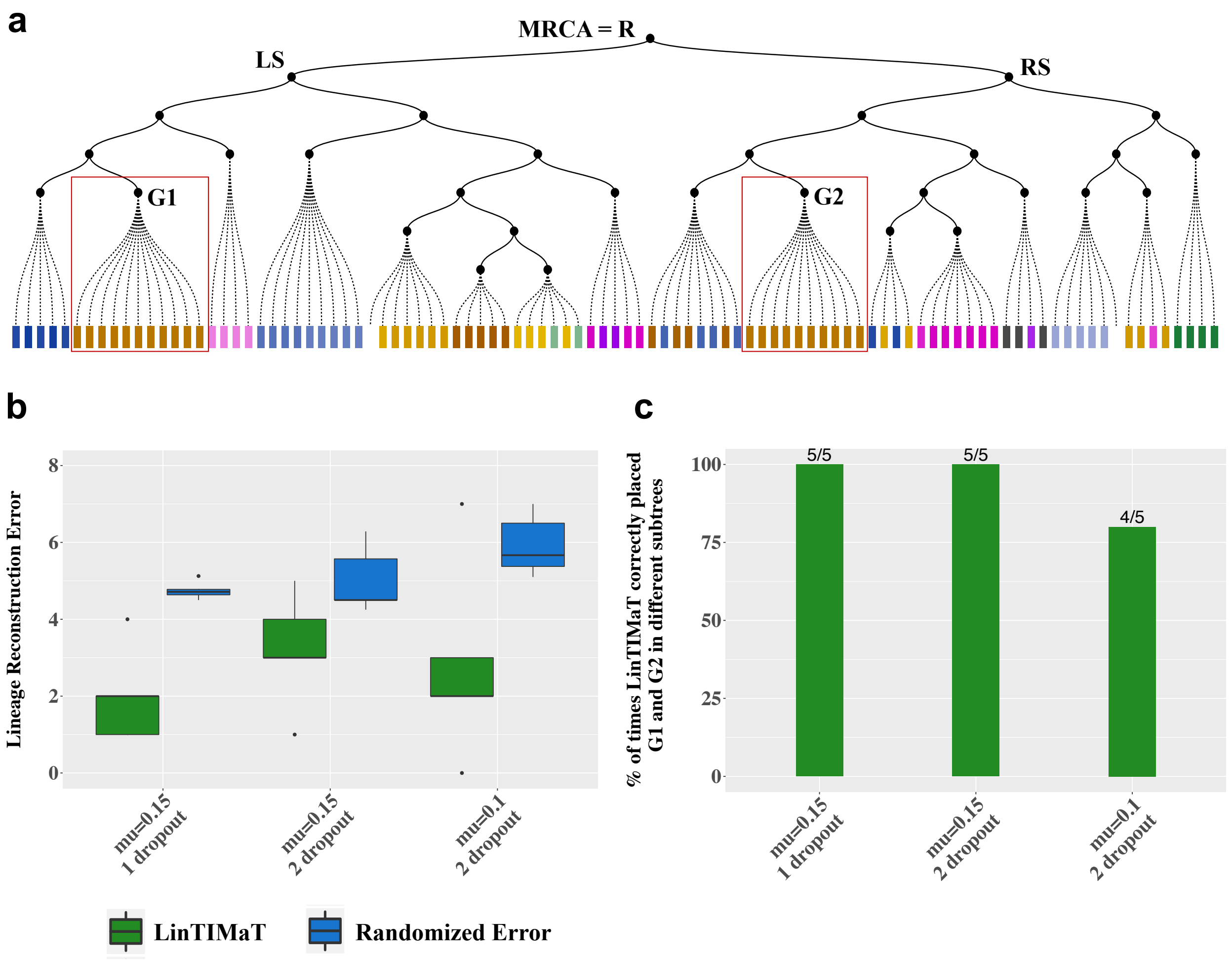

Supplementary Figure 6: Performance of LinTIMaT in recovering lineage relationship between cells undergoing convergent differentiation when no CRISPR mutations are shared between the groups of cells. (a) An example simulated lineage. G1 and G2 are the groups of cells that have distinct developmental history (root of lineage being their most recent common ancestor, MRCA) but converged to similar transcriptional state (same cell type). G1 is present in the left subtree (LS) and G2 is present in the right subtree (RS). See Supplementary Figure 10 for cell type color legend. (b) Performance of LinTIMaT in recovering the lineage between G1 and G2. LinTIMaT's lineage reconstruction error is compared against a randomized error that represents the average lineage reconstruction error considering the case when G1 and G2 are placed in the same subtree. Each box-and-whisker plot summarizes results for 5 replicates, where the box shows the interquartile range (IQR, the range between the 25th and 75th percentile) with the median value, whiskers indicate the maximum and minimum value within 1.5 times the IQR, also shown are outliers as black dots. (c) Performance of LinTIMaT in placing G1 and G2 in two different subtrees under different experimental conditions.

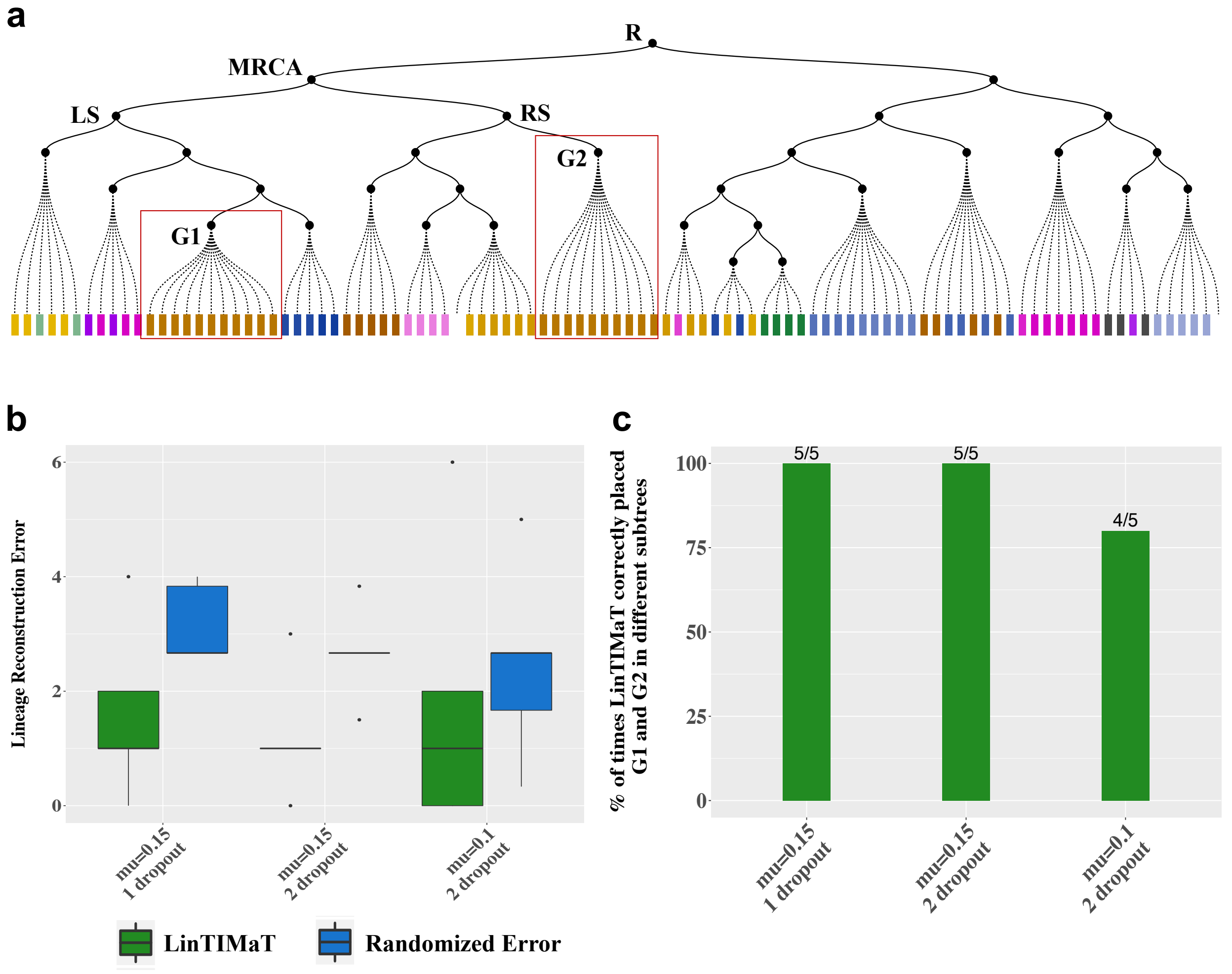

Supplementary Figure 7: Performance of LinTIMaT in recovering lineage relationship between cells undergoing convergent differentiation when some CRISPR mutations are possibly shared between the groups of cells. (a) An example simulated lineage. G1 and G2 are the groups of cells that converged to the same cell type but had distinct origin in the lineage (their most recent common ancestor, MRCA is a child of the root). G1 is present in the left subtree (LS) and G2 is present in the right subtree (RS). See Supplementary Figure 10 for cell type color legend. (b) Performance of LinTIMaT in recovering the lineage between G1 and G2 that show convergent differentiation. LinTIMaT's lineage reconstruction error is compared against a randomized error that represents the average lineage reconstruction error considering the case when G1 and G2 are placed in the same subtree. Each box-and-whisker plot summarizes results for 5 replicates, where the box shows the interquartile range (IQR, the range between the 25th and 75th percentile) with the median value, whiskers indicate the maximum and minimum value within 1.5 times the IQR, also shown are outliers as black dots. (c) Performance of LinTIMaT in placing G1 and G2 in two different subtrees under different experimental conditions.

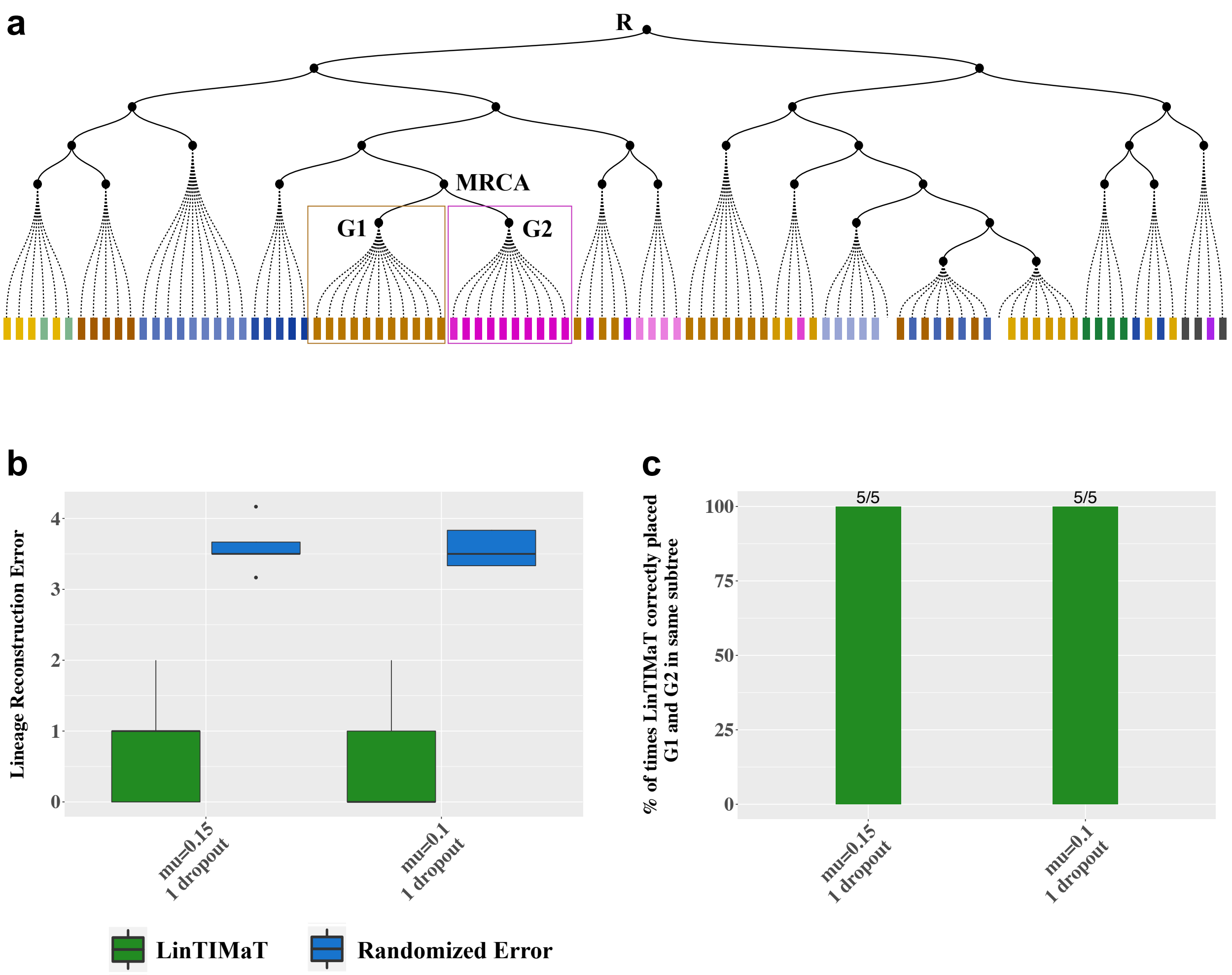

Supplementary Figure 8: Performance of LinTIMaT in recovering lineage relationship between two groups of cells that underwent divergent differentiation where the cells are transcriptionally distinct (different cell type) but have a common ancestry. (a) An example simulated lineage. G1 and G2 are the groups of cells that are from different cell types (neuron and progenitor) but they share the same lineage and are next to each other, parent of G1 and G2 is their most recent common ancestor (MRCA). See Supplementary Figure 10 for cell type color legend. (b) Performance of LinTIMaT in recovering the lineage relationship between G1 and G2. LinTIMaT's lineage reconstruction error is compared against a randomized error that represents the average lineage reconstruction error considering the case when G1 and G2 are placed in different subtrees. Each box-and-whisker plot summarizes results for 5 replicates, where the box shows the interquartile range (IQR, the range between the 25th and 75th percentile) with the median value, whiskers indicate the maximum and minimum value within 1.5 times the IQR, also shown are outliers as black dots. (c) Performance of LinTIMaT in placing G1 and G2 in the same subtree under different experimental conditions.

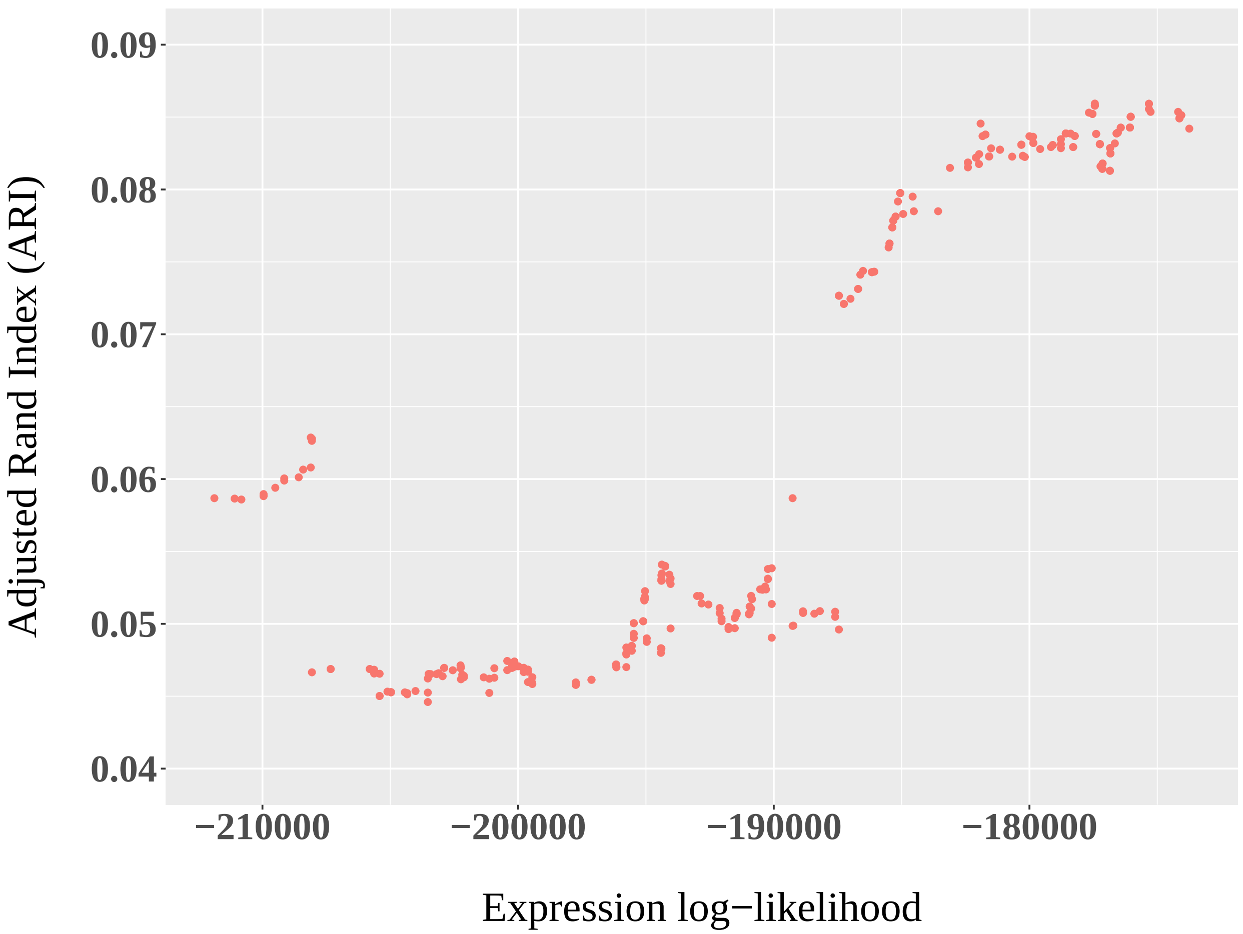

Supplementary Figure 9: Adjusted Rand Index (ARI) which measures the agreement between cell types in the tree clusters and cell types assigned by the original paper [5] as a function of the likelihood computed by LinTIMaT for ZF1. The fact that as the likelihood increases the ARI increases as well indicates that the target function of LinTIMaT is capturing biologically relevant relationships between cells.

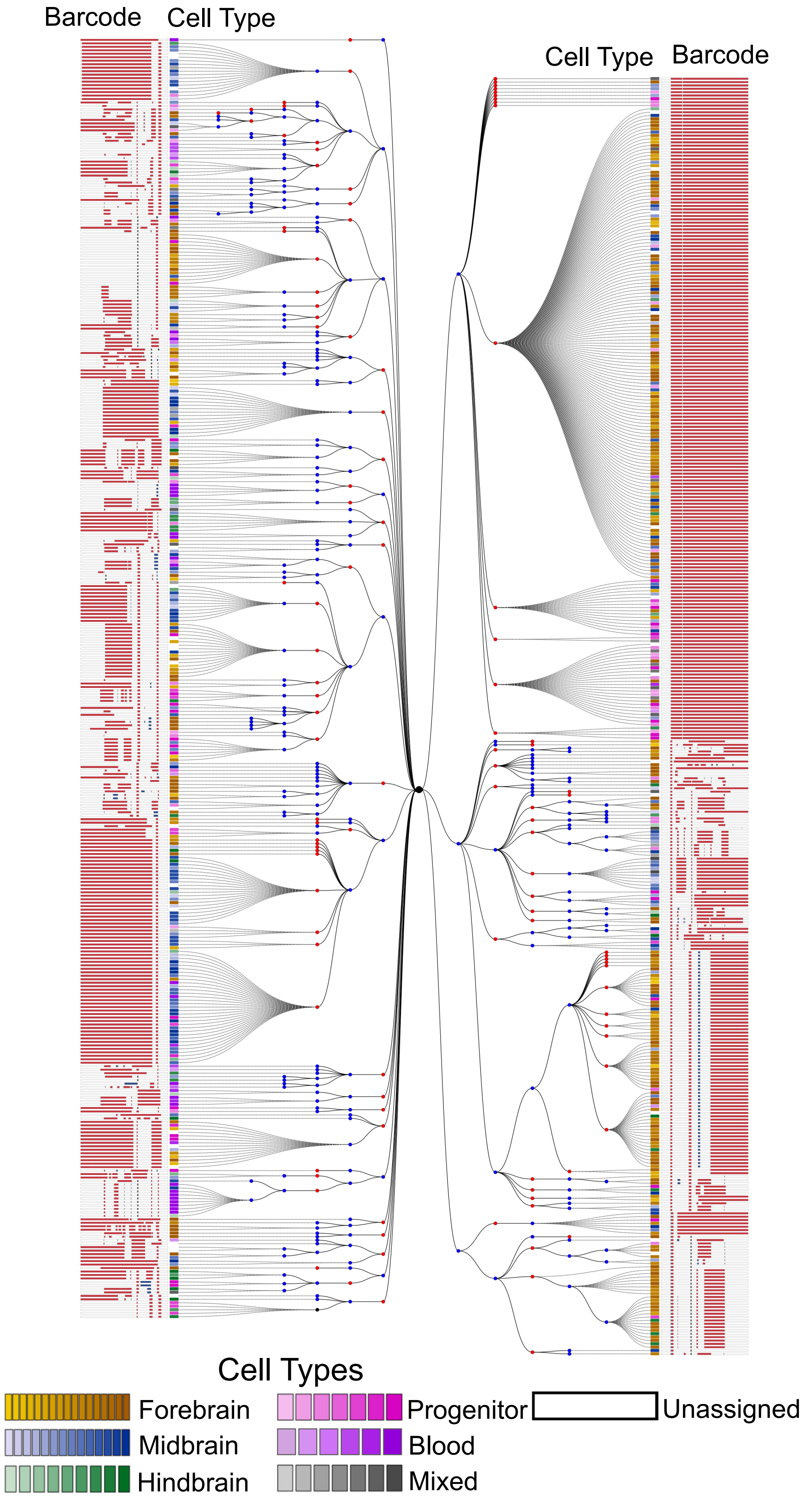

Supplementary Figure 10: The lineage tree reconstructed by LinTIMaT from a single juvenile zebrafish brain (ZF1) dataset generated by scGESTALT. The lineage tree is built on 750 cells. Blue nodes represent Cas9-editing events (mutations) and red nodes represent clusters inferred by LinTIMaT from transcriptomic data. Each leaf node is a cell, represented by a square, and its color represents its cell type as indicated in the legend. The mutated barcode for each cell is displayed as a white bar with insertions (blue) and deletions (red).

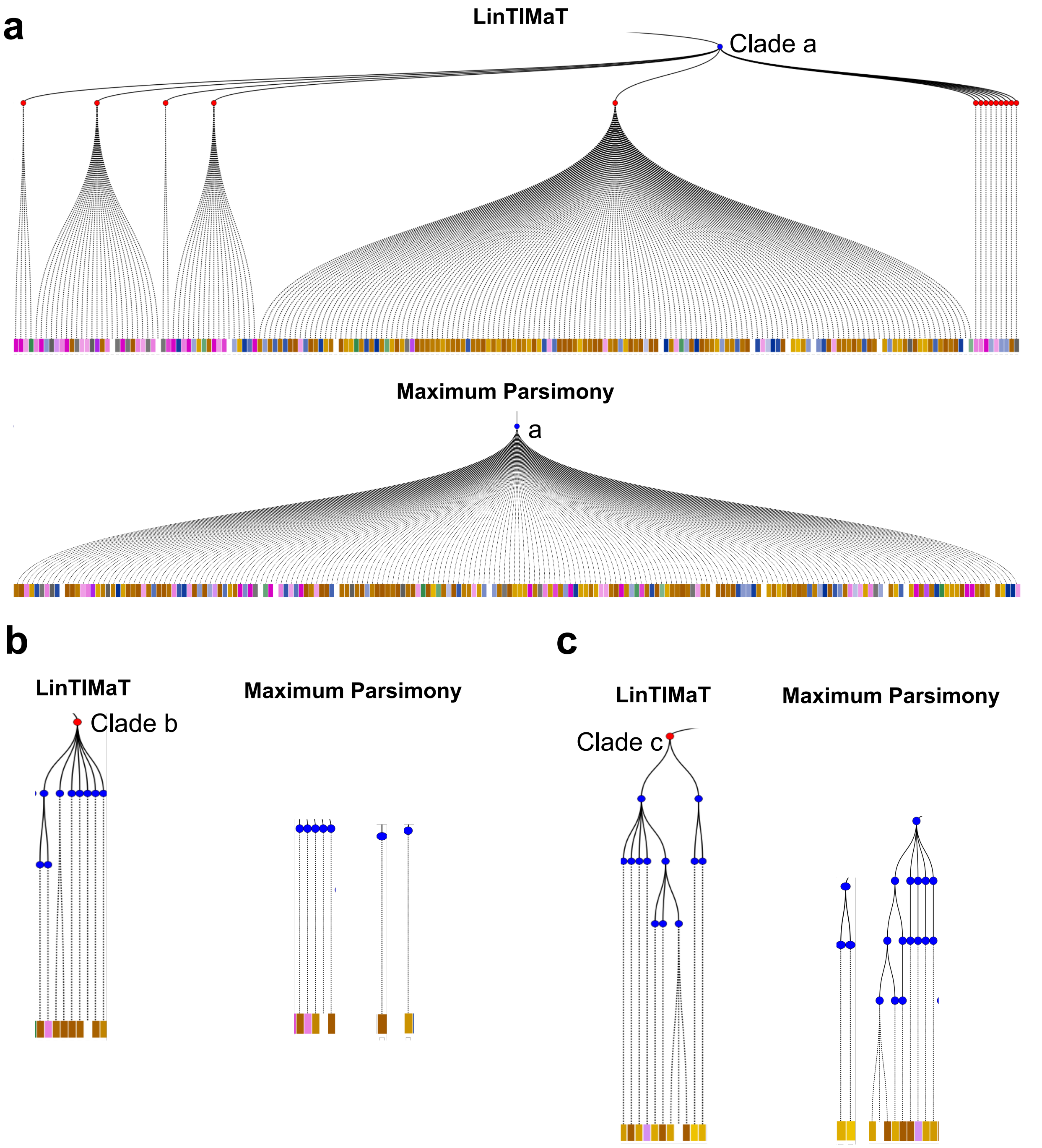

Supplementary Figure 11: Example subtrees in the lineage tree reconstructed by LinTIMaT from a single juvenile zebrafish brain (ZF1) dataset generated by scGESTALT. (a) Example subtree showing ability of LinTIMaT in separating cells with exactly the same barcode to distinct clusters of cell types. (b-c) Example subtrees displaying LinTIMaT's ability to cluster cells with different barcodes together based on their cell types, maximum parsimony puts them on distinct branches.

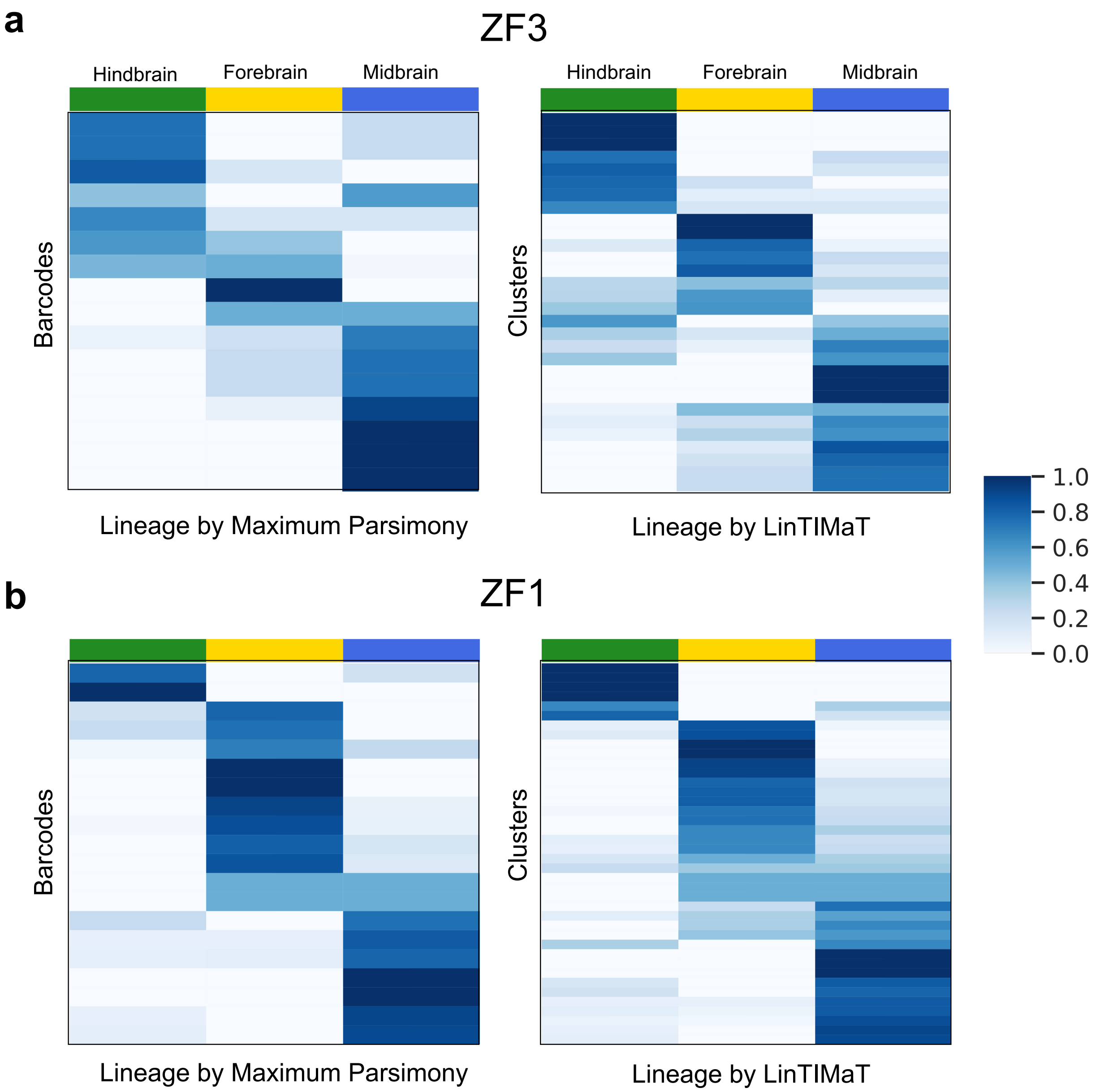

Supplementary Figure 12: Distribution of cell types in the juvenile zebrafish brain for scGESTALT datasets. Heat map of the distribution of cell clusters for each region of the brain (columns). Cell types were classified as belonging to the forebrain, midbrain or hindbrain, and the proportions of cells within each region were calculated for each cluster. For MP lineage, the rows of the heat map represent barcodes, for LinTIMaT lineage, the rows represent clusters inferred from barcodes and expression data. (a) Comparison for ZF3. (b) Comparison for ZF1.

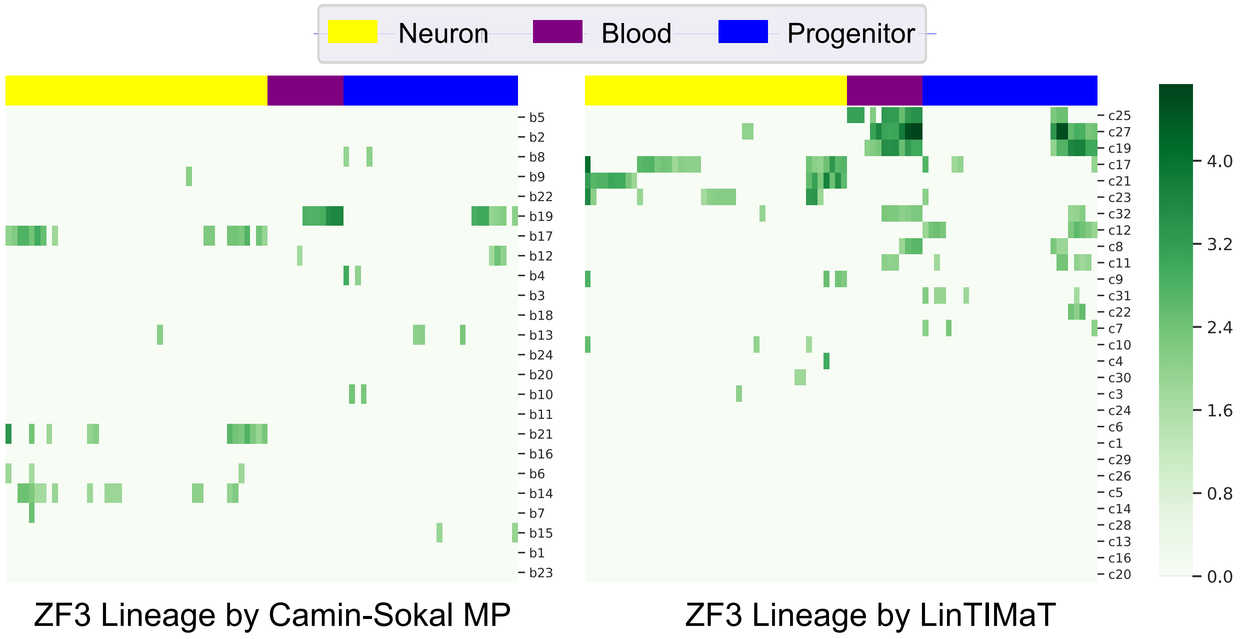

Supplementary Figure 13: Comparison of GO analysis for lineage trees reconstructed by Camin-Sokal Maximum Parsimony and LinTIMaT for a single juvenile zebrafish brain (ZF3) dataset generated by scGESTALT. The figure shows heat map of the square rooted negative log p-values of all GO terms for the clusters in the reconstructed lineage. The significant GO terms are obtained from g:Profiler [17], where hypergeometric distribution is adopted for performing statistical test and the p-values are adjusted based on the g:SCS algorithm [18]. The rows represent clusters and the columns represent different GO terms as shown in Supplementary Table. The values were colored as shown in the key. The yellow, purple and blue columns correspond to GO terms related to neurons, blood and progenitors respectively. The left panel shows the heat map for the barcode clusters in MP reconstructed lineage, and the right panel shows the heat map for the clusters in LinTIMaT reconstructed lineage.

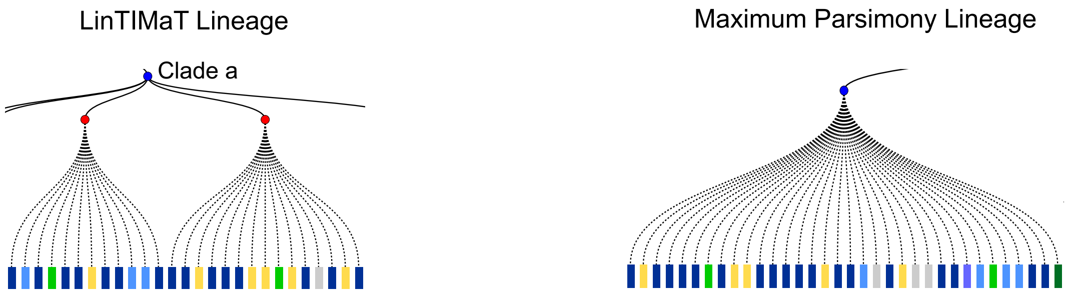

Supplementary Figure 14: Example subtree in the lineage tree reconstructed by LinTIMaT for a zebrafish dataset (R2) generated using ScarTrace. This subtree shows the ability of LinTIMaT in separating cells with exactly the same barcode to distinct clusters of cell types. See Supplementary Figure 15 for cell type color legend.

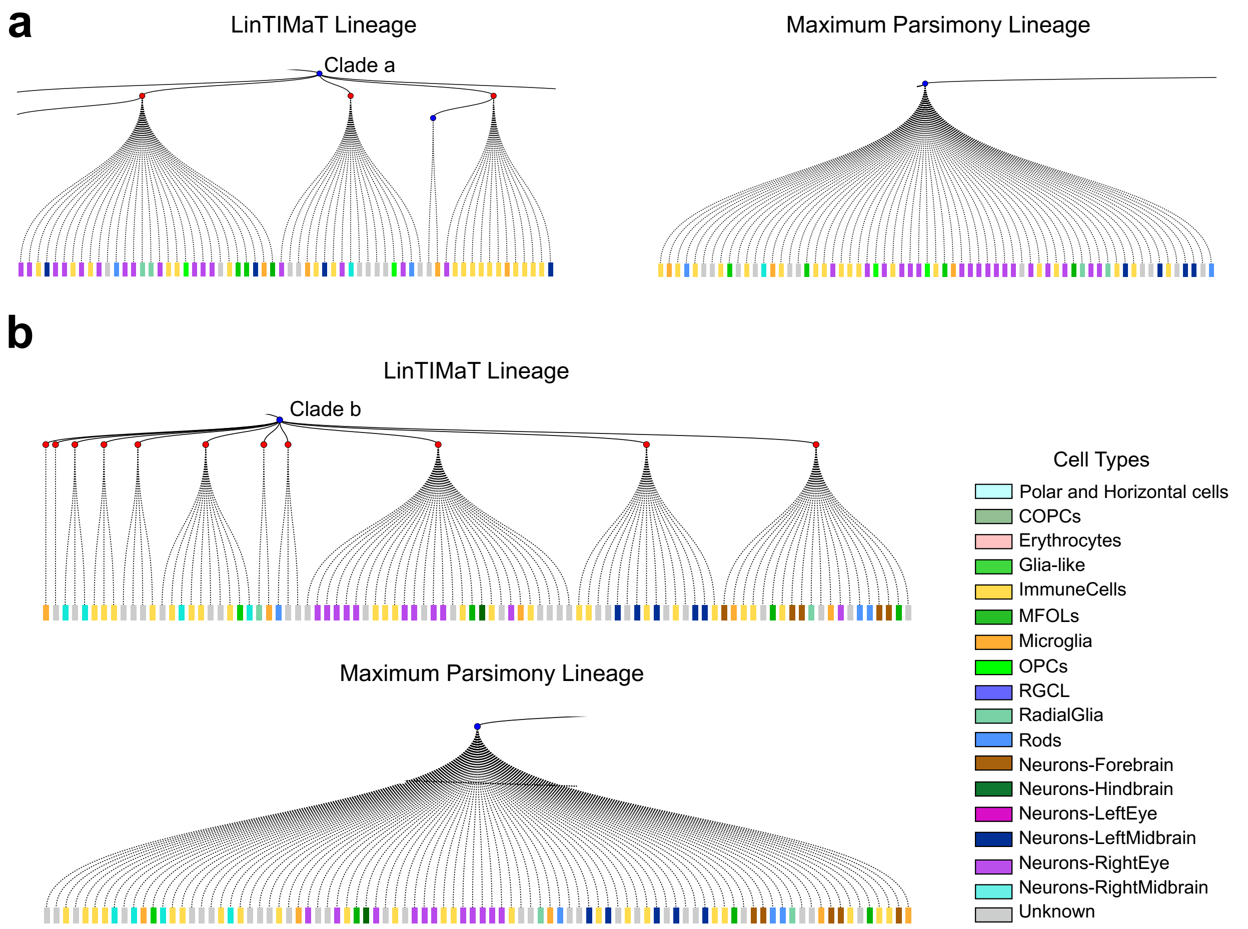

Supplementary Figure 15: Example subtrees in the lineage tree reconstructed by LinTIMaT for a zebrafish dataset (R3) generated using ScarTrace. These subtrees illustrate the ability of LinTIMaT in separating cells with exactly the same barcode to distinct clusters of cell types.

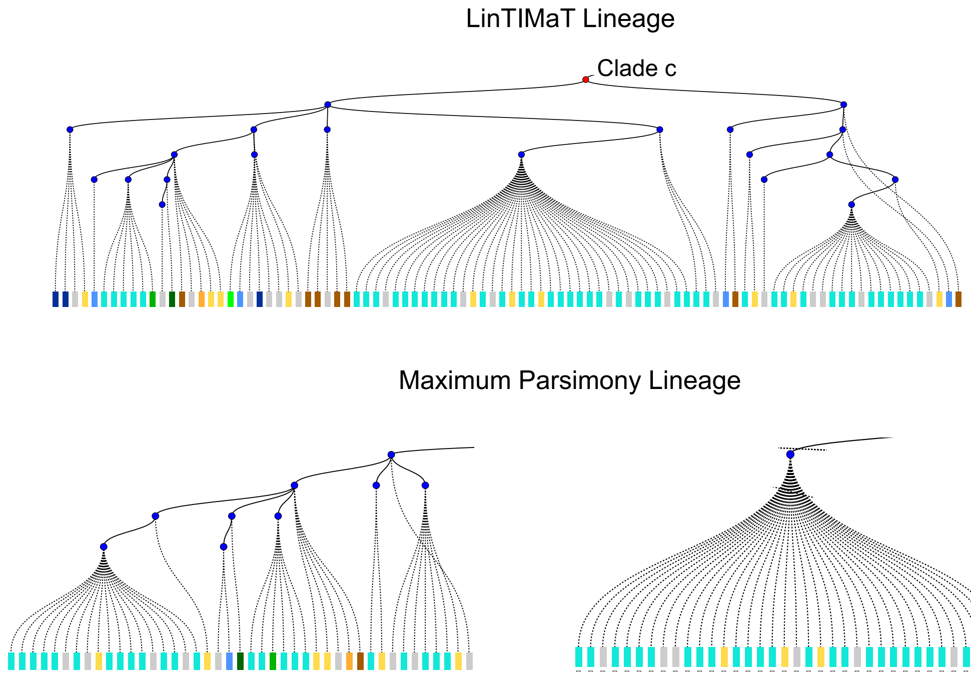

Supplementary Figure 16: Example subtree in the lineage tree reconstructed by LinTIMaT for a zebrafish dataset (R3) generated using ScarTrace. This subtree displays LinTIMaT's ability to cluster cells with different barcodes together based on their cell types, maximum parsimony puts them on distinct branches. See Supplementary Figure 15 for cell type color legend.

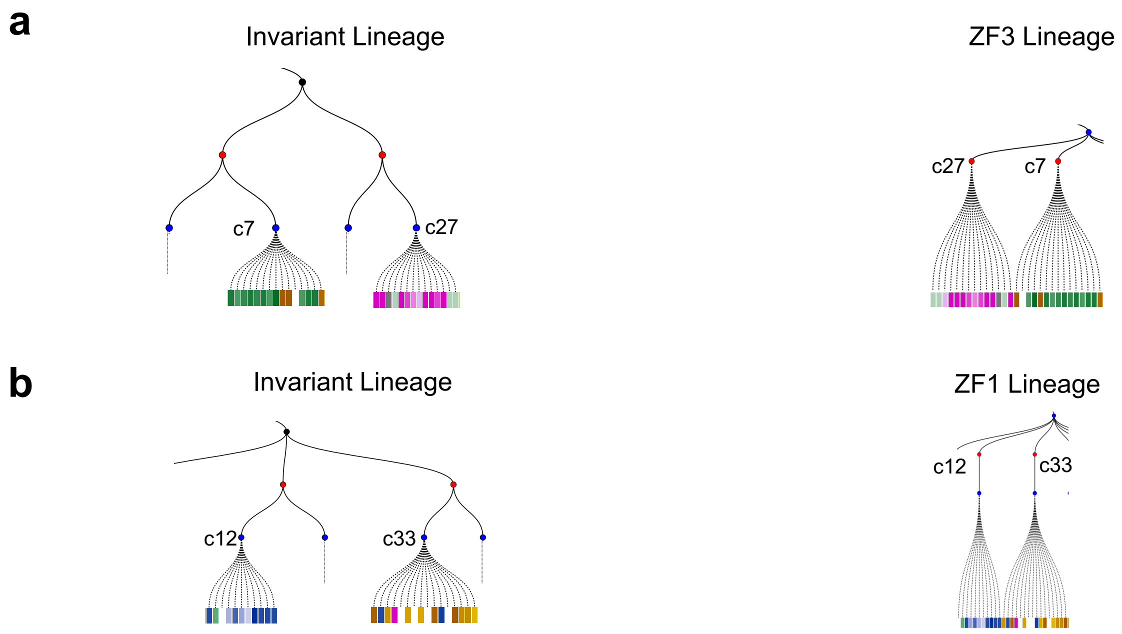

Supplementary Figure 17: Invariant lineage preserves ancestor-descendant relationships in individual lineages reconstructed for scGESTALT datasets. (a) Clusters c7 and c27 are present in the same subtree in both the invariant lineage and ZF3 lineage. (b) Clusters c12 and c33 are present in the same subtree in both the invariant lineage and ZF1 lineage.

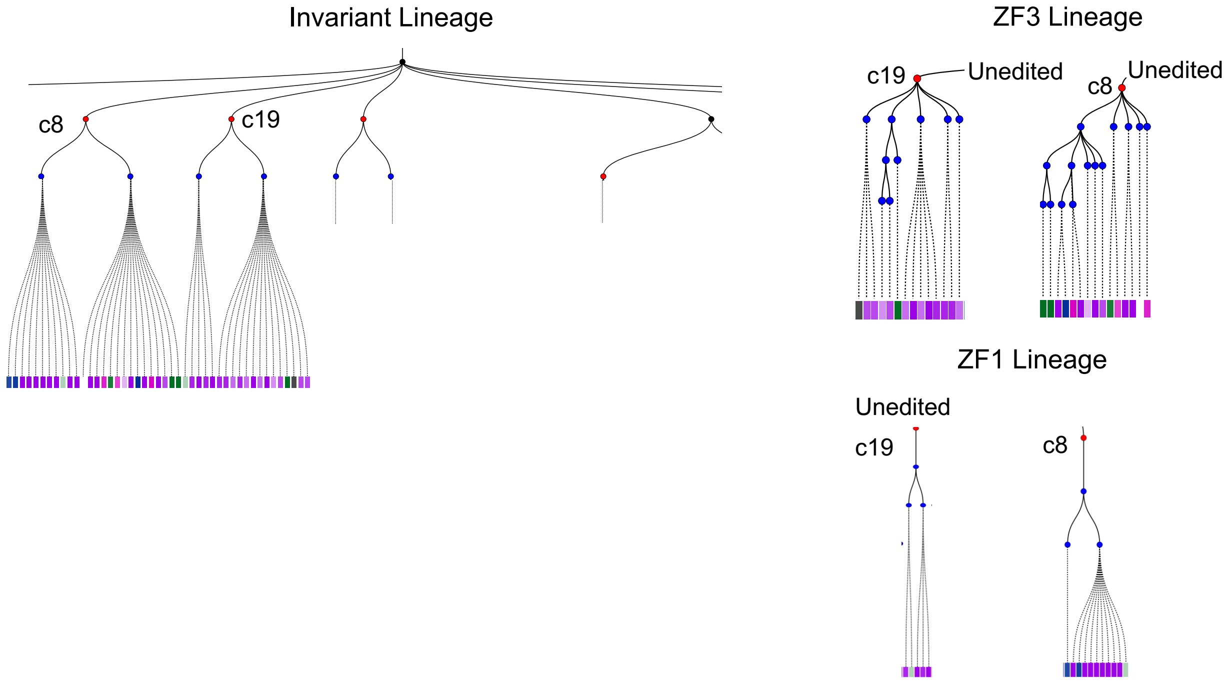

Supplementary Figure 18: Invariant lineage places similar cell clusters together in the same subtree. In ZF3 (generated by scGESTALT) lineage, clusters c8 and c19 both contain cells belonging to blood cell type but these clusters are placed in different branches. In invariant lineage these clusters are placed in the same subtree. Similar examples are observed for ZF1 lineage.

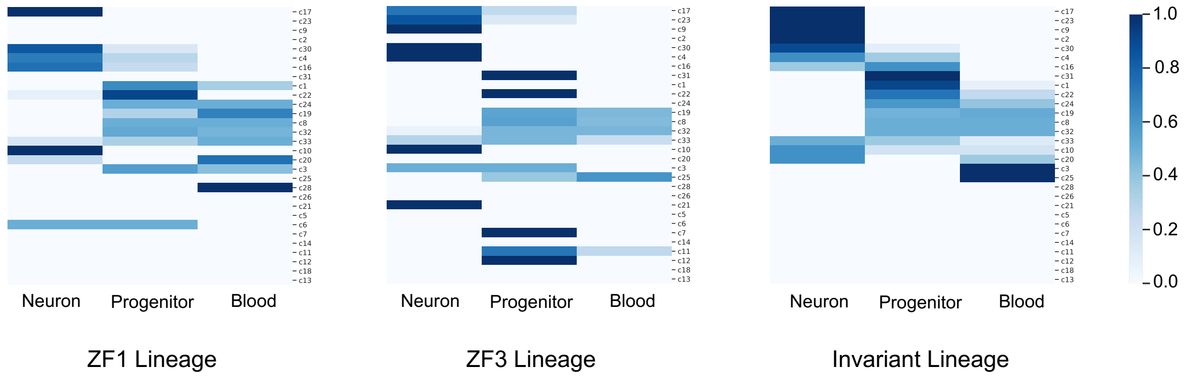

Supplementary Figure 19: Proportions of each type of GO terms for the invariant clusters (for scGESTALT dataset). The rows represent invariant clusters and the columns represent different types of GO terms.

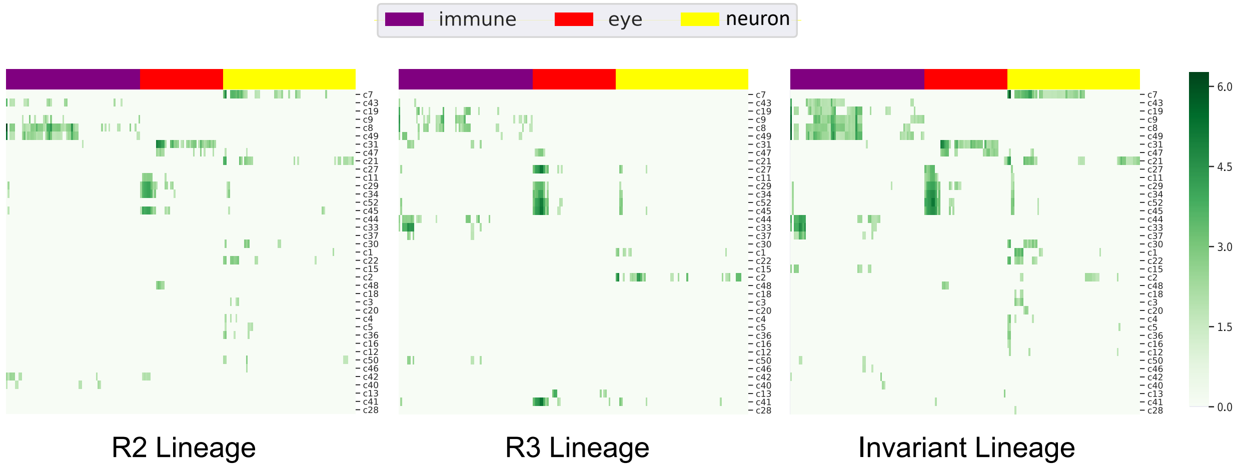

Supplementary Figure 20: Heat map of the square rooted negative log p-values of all GO terms for the invariant clusters that contains 10 or more cells for the ScarTrace dataset. The significant GO terms were obtained from g:Profiler [17], where hypergeometric distribution is adopted for performing statistical test and the p-values are adjusted based on the g:SCS algorithm [18]. The rows represent selected invariant clusters and the columns represent different GO terms as shown in Supplementary Table S9. The values were colored as shown in the key. The yellow, purple and red columns correspond to GO terms related to neurons, immune celltype and eye respectively. The leftmost panel shows the heat map for the clusters in R2 lineage, middle panel shows the heat map for R3 lineage and the righmost panel shows the heat map for the invariant lineage.

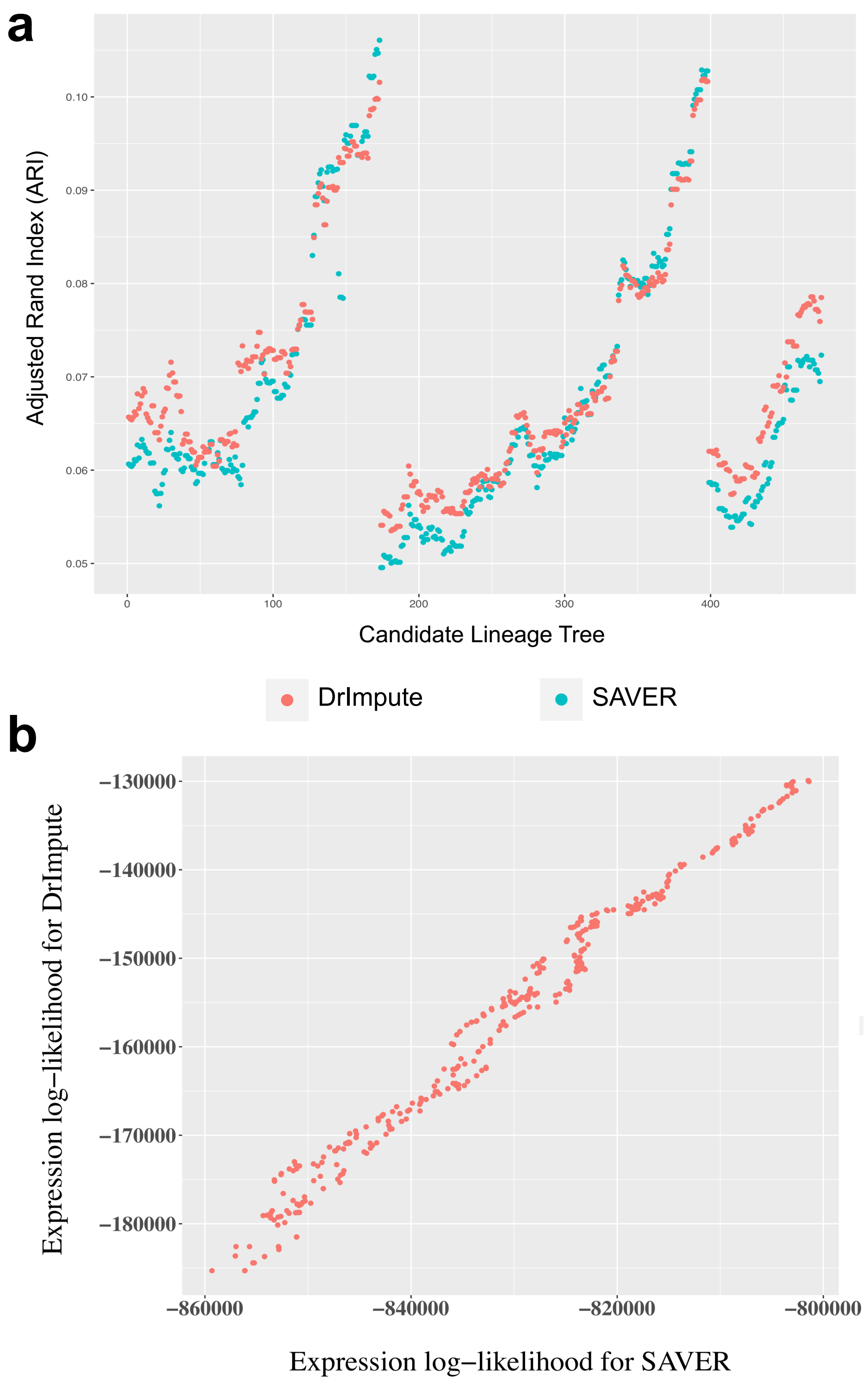

Supplementary Figure 21: (a) Effect of the imputation method on LinTIMaT's expression likelihood function displayed through cell clustering performance. For a set of candidate lineage trees for ZF3, we compared the cell clustering based on expression likelihood for expression data imputed using two imputation methods: DrImpute and SAVER. The cell clustering performance is measured in terms of Adjusted Rand Index. (b) Plot comparing the expression log-likelihoods for a set of lineage trees for data imputed using DrImpute and SAVER. Correlation 0.9914.

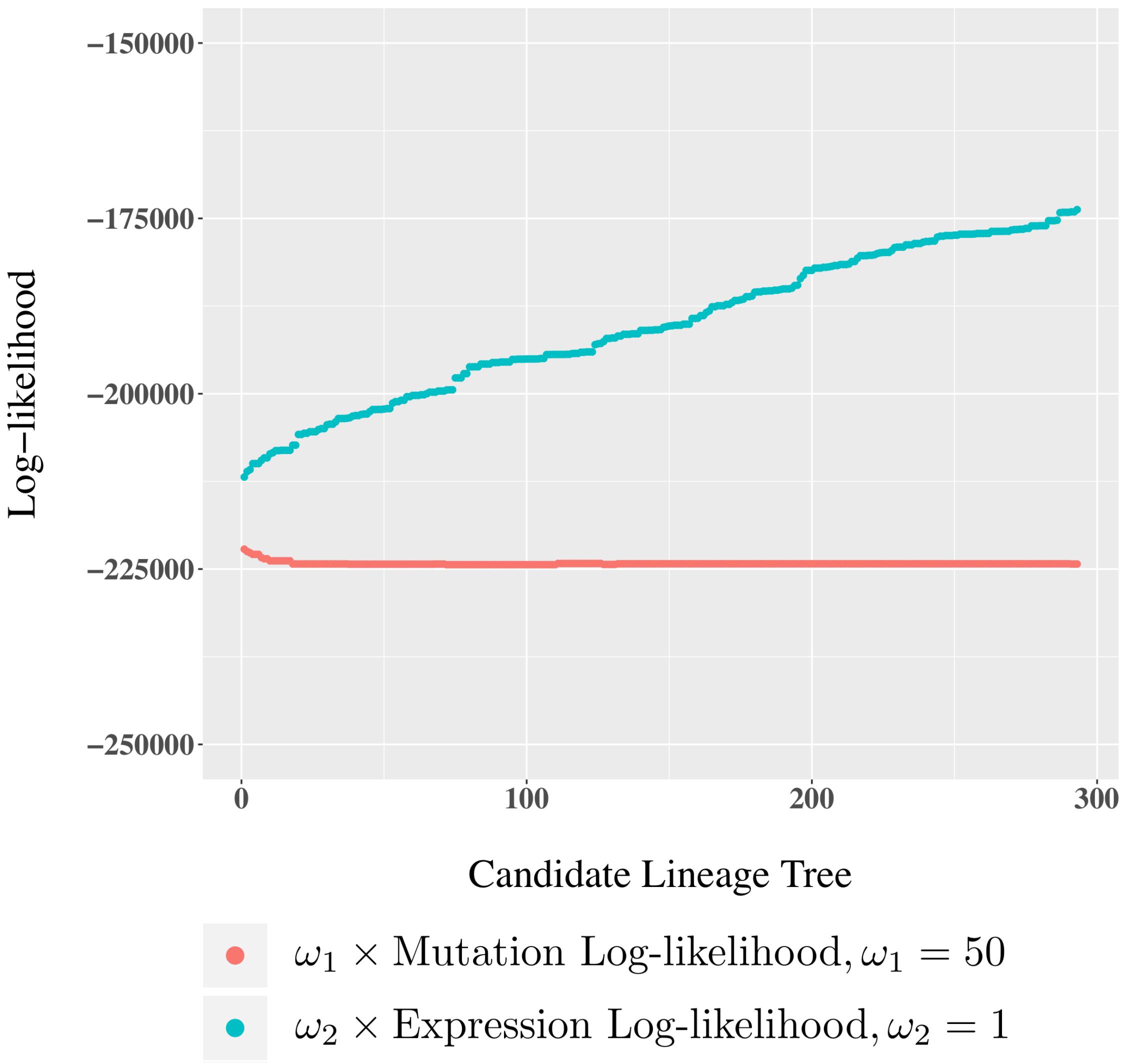

Supplementary Figure 22: Comparison of weighted values of mutation log-likelihood and expression log-likelihood for specific weights, $\omega_1 = 50$ and $\omega_2 = 1$ for a set of candidate lineage trees for ZF1. For these values of weights, the weighted values of the two log-likelihoods remain in the same range.

4 Supplementary Tables

Supplementary Table 1: The ARI for each scGESTALT tree calculated based on different levels

ZF1			ZF3		
Level	#cluster	ARI	Level	#cluster	ARI
1	25	0.035658001	1	23	0.01938034
2	75	0.047930182	2	67	0.037909748
3	363	0.041749244	3	153	0.045092761
4	531	0.06103636	4	279	0.027522376
5	676	0.04716886	5	368	0.002327659
6	750	0	6	376	0
Barcode	192	0.061237582	Barcode	150	0.056133354

Supplementary Table 2: Comparison of log-likelihood score of lineage trees for scGESTALT datasets based on only mutation data

Method	ZF1	ZF3
LinTIMaT	−4485.564873	−2871.118661
MP	−303463.075241	−102474.127300

Supplementary Table 3: Comparison of log-likelihood score of lineage trees for ScarTrace datasets based on only mutation data

Method	R2	R3
LinTIMaT	−2111.155680	−1816.091048
MP	−501741.626610	−301271.047508

Supplementary Table 4: Strings for filtering the GO terms for each GO type for the scGESTALT dataset

**GO type**	**filter strings**
neuron	neuro nervous synap
blood	heme hema hemo erythrocyte myeloid hscs immune
progenitor	develop differentiat

Supplementary Table 5: Strings for filtering the GO terms for each GO type for the ScarTrace dataset

**GO type**	**filter strings**
neuron	neuro nervous synap
immune	heme hema hemo erythrocyte myeloid hscs immune
eye	photoreceptor retina eye phototransduction optic visual light_stimulus

Supplementary Table 6: Full list of GO terms and corresponding p-values for scGESTALT ZF3 appearing in LinTIMaT clusters but not in any individual clusters for MP tree. The significant GO terms were obtained from g:Profiler [17], where hypergeometric distribution is adopted for performing statistical test and the p-values are adjusted based on g:SCS algorithm [18].

**GO term**	**cluster, p-value**
Acetylcholine Neurotransmitter Release Cycle	(c23,1.49e-02)
Norepinephrine Neurotransmitter Release Cycle	(c23,1.11e-02)
neuron development	(c17,4.41e-04)
heme-copper terminal oxidase activity	(c25,5.87e-05)
neurotransmitter receptor complex	(c23,1.07e-02)
neurogenesis	(c17,1.26e-02)
oxidoreductase activity, acting on a heme group of donors, oxygen as acceptor	(c25,5.87e-05)
Serotonin Neurotransmitter Release Cycle	(c23,1.11e-02)
Optic neuropathy	(c10,1.98e-02)
presynaptic cytoskeleton	(c30,3.75e-02)
postsynaptic density	(c23,4.98e-02)
oxidoreductase activity, acting on a heme group of donors	(c25,5.87e-05)
animal organ development	(c27,5.08e-03),(c19,1.24e-04),(c11,2.45e-02),(c12,8.33e-03)
cytoskeleton of presynaptic active zone	(c30,3.75e-02)
peripheral nervous system neuron axonogenesis	(c32,2.66e-02)
peripheral nervous system neuron differentiation	(c27,1.90e-02)
Hematological neoplasm	(c27,5.56e-05),(c19,9.13e-03),(c25,9.13e-03)
peripheral nervous system neuron development	(c27,1.90e-02)
generation of neurons	(c17,3.43e-03)
postsynapse	(c23,1.21e-02)
regulation of cell differentiation	(c31,3.40e-02)
cell development	(c17,2.08e-02)
neuron differentiation	(c23,2.82e-02),(c17,9.02e-04)
Global developmental delay	(c17,4.05e-02)
neuron projection development	(c17,5.39e-03)
immune system process	(c19,1.47e-02)
Neurological speech impairment	(c17,5.80e-04)

Supplementary Table 7: Full list of filtered GO terms used in GO p-value/proportion heat maps for the scGESTALT dataset individual tree of ZF3, see Supplementary Table 4 for the keywords we used to filter these GO terms.

index	GO type	GO term	index	GO type	GO term
1	neuron	neuron_synapse part	45	neuron	neuron_anterograde trans-synaptic signaling
2	neuron	neuron_postsynaptic density	46	blood	blood_heme-copper terminal oxidase activity
3	neuron	neuron_neuronal ion channel clustering	47	blood	blood_erythrocyte homeostasis
4	neuron	neuron_chemical synaptic transmission	48	blood	blood_hemopoiesis
5	neuron	neuron_trans-synaptic signaling	49	blood	blood_Hematological neoplasm
6	neuron	neuron_neuron maturation	50	blood	blood_Abnormal erythrocyte morphology
7	neuron	neuron_vesicle-mediated transport in synapse	51	blood	blood_immune system development
8	neuron	neuron_postsynapse	52	blood	blood_oxidoreductase activity acting on a heme group of donors oxygen as acceptor
9	neuron	neuron_synaptic vesicle membrane	53	blood	blood_hematopoietic or lymphoid organ development
10	neuron	neuron_Acetylcholine Neurotransmitter Release Cycle	54	blood	blood_myeloid cell differentiation
11	neuron	neuron_Neurotransmitter release cycle	55	blood	blood_myeloid cell homeostasis
12	neuron	neuron_synaptic vesicle	56	blood	blood_oxidoreductase activity acting on a heme group of donors
13	neuron	neuron_neurotransmitter secretion	57	blood	blood_erythrocyte differentiation
14	neuron	neuron_neuron projection	58	blood	blood_immune system process
15	neuron	neuron_Norepinephrine Neurotransmitter Release Cycle	59	progenitor	progenitor_multicellular organism development
16	neuron	neuron_Glutamate Neurotransmitter Release Cycle	60	progenitor	progenitor_nervous system development
17	neuron	neuron_generation of neurons	61	progenitor	progenitor_cell development
18	neuron	neuron_neurotransmitter receptor complex	62	progenitor	progenitor_brain development
19	neuron	neuron_synaptic signaling	63	progenitor	progenitor_embryo development ending in birth or egg hatching
20	neuron	neuron_synapse	64	progenitor	progenitor_anatomical structure development
21	neuron	neuron_Neurological speech impairment	65	progenitor	progenitor_animal organ development
22	neuron	neuron_regulation of neurotransmitter levels	66	progenitor	progenitor_embryo development
23	neuron	neuron_neuron part	67	progenitor	progenitor_immune system development
24	neuron	neuron_synaptic vesicle cycle	68	progenitor	progenitor_posterior lateral line development
25	neuron	neuron_neurogenesis	69	progenitor	progenitor_peripheral nervous system neuron differentiation
26	neuron	neuron_peripheral nervous system neuron differentiation	70	progenitor	progenitor_hematopoietic or lymphoid organ development
27	neuron	neuron_presynapse	71	progenitor	progenitor_hindbrain development
28	neuron	neuron_peripheral nervous system neuron development	72	progenitor	progenitor_peripheral nervous system neuron development
29	neuron	neuron_synaptic vesicle exocytosis	73	progenitor	progenitor_central nervous system development
30	neuron	neuron_Dopamine Neurotransmitter Release Cycle	74	progenitor	progenitor_transdifferentiation
31	neuron	neuron_neuron development	75	progenitor	progenitor_myeloid cell differentiation
32	neuron	neuron_Neurotransmitter uptake and metabolism In glial cells	76	progenitor	progenitor_neuron development
33	neuron	neuron_neurotransmitter transport	77	progenitor	progenitor_head development
34	neuron	neuron_posterior lateral line neuromast development	78	progenitor	progenitor_posterior lateral line system development
35	neuron	neuron_Transmission across Chemical Synapses	79	progenitor	progenitor_mechanosensory lateral line system development
36	neuron	neuron_Optic neuropathy	80	progenitor	progenitor_posterior lateral line neuromast development
37	neuron	neuron_cytoskeleton of presynaptic active zone	81	progenitor	progenitor_Global developmental delay
38	neuron	neuron_peripheral nervous system neuron axonogenesis	82	progenitor	progenitor_developmental process
39	neuron	neuron_signal release from synapse	83	progenitor	progenitor_regulation of cell differentiation
40	neuron	neuron_neuron differentiation	84	progenitor	progenitor_system development
41	neuron	neuron_Neuronal System	85	progenitor	progenitor_neuron differentiation
42	neuron	neuron_neuron projection development	86	progenitor	progenitor_neuron projection development
43	neuron	neuron_Serotonin Neurotransmitter Release Cycle	87	progenitor	progenitor_erythrocyte differentiation
44	neuron	neuron_presynaptic cytoskeleton	88	progenitor	progenitor_chordate embryonic development

Supplementary Table 8: Full list of filtered GO terms used in GO p-value/proportion heat maps for the scGESTALT dataset invariant tree, see Supplementary Table 4 for the keywords we used to filter these GO terms.

index	GO type	GO term	index	GO type	GO term
1	neuron	neuron_synapse part	58	blood	blood_erythrocyte homeostasis
2	neuron	neuron_neuronal ion channel clustering	59	blood	blood_hemopoiesis
3	neuron	neuron_chemical synaptic transmission	60	blood	blood_Abnormality of the immune system
4	neuron	neuron_neuron maturation	61	blood	blood_myeloid cell development
5	neuron	neuron_vesicle-mediated transport in synapse	62	blood	blood_Abnormal cellular immune system morphology
6	neuron	neuron_postsynapse	63	blood	blood_Hematological neoplasm
7	neuron	neuron_neuron projection guidance	64	blood	blood_Abnormal erythrocyte morphology
8	neuron	neuron_postsynaptic density	65	blood	blood_immune system development
9	neuron	neuron_peripheral nervous system neuron development	66	blood	blood_oxidoreductase activity, acting on a heme group of donors, oxygen as acceptor
10	neuron	neuron_synaptic vesicle membrane	67	blood	blood_hematopoietic or lymphoid organ development
11	neuron	neuron_neuron projection cytoplasm	68	blood	blood_myeloid cell differentiation
12	neuron	neuron_Neurotransmitter release cycle	69	blood	blood_myeloid cell homeostasis
13	neuron	neuron_Acetylcholine Neurotransmitter Release Cycle	70	blood	blood_Cytokine Signaling in Immune system
14	neuron	neuron_synaptic signaling	71	blood	blood_definitive hemopoiesis
15	neuron	neuron_neurotransmitter secretion	72	blood	blood_oxidoreductase activity, acting on a heme group of donors
16	neuron	neuron_neuron projection	73	blood	blood_erythrocyte differentiation
17	neuron	neuron_Norepinephrine Neurotransmitter Release Cycle	74	blood	blood_immune system process
18	neuron	neuron_negative regulation of neuron differentiation	75	progenitor	progenitor_multicellular organism development
19	neuron	neuron_neuronal cell body	76	progenitor	progenitor_brain development
20	neuron	neuron_generation of neurons	77	progenitor	progenitor_cellular developmental process
21	neuron	neuron_presynaptic active zone	78	progenitor	progenitor_Delayed speech and language development
22	neuron	neuron_neurotransmitter receptor complex	79	progenitor	progenitor_RUNX1 regulates genes involved in megakaryocyte differentiation and platelet function
23	neuron	neuron_Neurodevelopmental delay	80	progenitor	progenitor_Developmental Biology
24	neuron	neuron_synapse	81	progenitor	progenitor_peripheral nervous system neuron development
25	neuron	neuron_Neurological speech impairment	82	progenitor	progenitor_erythrocyte development
26	neuron	neuron_regulation of neurotransmitter levels	83	progenitor	progenitor_cell development
27	neuron	neuron_neuron part	84	progenitor	progenitor_embryo development ending in birth or egg hatching
28	neuron	neuron_synaptic vesicle cycle	85	progenitor	progenitor_anatomical structure development
29	neuron	neuron_neurogenesis	86	progenitor	progenitor_myeloid cell development
30	neuron	neuron_peripheral nervous system neuron differentiation	87	progenitor	progenitor_nervous system development
31	neuron	neuron_presynapse	88	progenitor	progenitor_negative regulation of neuron differentiation
32	neuron	neuron_synaptic vesicle	89	progenitor	progenitor_positive regulation of developmental process
33	neuron	neuron_postsynaptic specialization membrane	90	progenitor	progenitor_animal organ development
34	neuron	neuron_synaptic vesicle exocytosis	91	progenitor	progenitor_tissue development
35	neuron	neuron_Dopamine Neurotransmitter Release Cycle	92	progenitor	progenitor_embryo development
36	neuron	neuron_neuron development	93	progenitor	progenitor_immune system development
37	neuron	neuron_Neurotransmitter uptake and metabolism In glial cells	94	progenitor	progenitor_Neurodevelopmental delay
38	neuron	neuron_neurotransmitter transport	95	progenitor	progenitor_Developmental regression
39	neuron	neuron_posterior lateral line neuromast development	96	progenitor	progenitor_peripheral nervous system neuron differentiation
40	neuron	neuron_Transmission across Chemical Synapses	97	progenitor	progenitor_hematopoietic or lymphoid organ development
41	neuron	neuron_Optic neuropathy	98	progenitor	progenitor_hindbrain development
42	neuron	neuron_cytoskeleton of presynaptic active zone	99	progenitor	progenitor_central nervous system development
43	neuron	neuron_peripheral nervous system neuron axonogenesis	100	progenitor	progenitor_regulation of developmental process
44	neuron	neuron_trans-synaptic signaling	101	progenitor	progenitor_myeloid cell differentiation
45	neuron	neuron_postsynaptic density membrane	102	progenitor	progenitor_head development
46	neuron	neuron_Glutamate Neurotransmitter Release Cycle	103	progenitor	progenitor_neuron development
47	neuron	neuron_signal release from synapse	104	progenitor	progenitor_Global developmental delay
48	neuron	neuron_neuron differentiation	105	progenitor	progenitor_posterior lateral line neuromast development
49	neuron	neuron_Neuronal System	106	progenitor	progenitor_developmental process
50	neuron	neuron_neuron projection development	107	progenitor	progenitor_regulation of cell differentiation
51	neuron	neuron_Polyneuropathy	108	progenitor	progenitor_neuron differentiation
52	neuron	neuron_Serotonin Neurotransmitter Release Cycle	109	progenitor	progenitor_neuron projection development
53	neuron	neuron_presynaptic cytoskeleton	110	progenitor	progenitor_cell differentiation
54	neuron	neuron_anterograde trans-synaptic signaling	111	progenitor	progenitor_system development
55	blood	blood_Abnormal immune system morphology	112	progenitor	progenitor_erythrocyte differentiation
56	blood	blood_heme-copper terminal oxidase activity	113	progenitor	progenitor_chordate embryonic development
57	blood	blood_erythrocyte development			

Supplementary Table 9: Full list of filtered GO terms used in GO p-value/proportion heat maps for the ScarTrace dataset, see Supplementary Table 5 for the keywords we used to filter these GO terms.

index	GO type	GO term	index	GO type	GO term
1	immune	immune_immune system process	100	eye	eye_sensory perception of light stimulus
2	immune	immune_Spontaneous hematomas	101	eye	eye_Aplasia/Hypoplasia of the optic nerve
3	immune	immune_hemopoiesis	102	eye	eye_Visual impairment
4	immune	immune_immune system development	103	eye	eye_photoreceptor cell development
5	immune	immune_hematopoietic or lymphoid organ development	104	eye	eye_Chorioretinal degeneration
6	immune	immune_erythrocyte homeostasis	105	eye	eye_photoreceptor cell differentiation
7	immune	immune_erythrocyte differentiation	106	eye	eye_Chorioretinal atrophy
8	immune	immune_myeloid cell differentiation	107	eye	eye_Attenuation of retinal blood vessels
9	immune	immune_myeloid cell homeostasis	108	eye	eye_Reduced visual acuity
10	immune	immune_Intestinal immune network for IgA production	109	eye	eye_photoreceptor cell outer segment organization
11	immune	immune_Abnormal lymphocyte morphology	110	eye	eye_Optic atrophy
12	immune	immune_Abnormal leukocyte count	111	eye	eye_Bone spicule pigmentation of the retina
13	immune	immune_Abnormal lymphocyte count	112	eye	eye_Abnormality of the optic disc
14	immune	immune_Abnormal lymphocyte physiology	113	eye	eye_Abnormal involuntary eye movements
15	immune	immune_leukocyte migration	114	eye	eye_Abnormal posterior eye segment morphology
16	immune	immune_myeloid leukocyte migration	115	eye	eye_Activation of the phototransduction cascade
17	immune	immune_leukocyte chemotaxis	116	eye	eye_Abnormality of the optic nerve
18	immune	immune_Innate Immune System	117	eye	eye_Optic disc hypoplasia
19	immune	immune_chemokine-mediated signaling pathway	118	eye	eye_Visual phototransduction
20	immune	immune_cellular response to chemokine	119	eye	eye_Retinal atrophy
21	immune	immune_response to chemokine	120	eye	eye_Cerebral visual impairment
22	immune	immune_Abnormality of the immune system	121	eye	eye_Optic disc pallor
23	immune	immune_macrophage chemotaxis	122	eye	eye_Abnormal conjugate eye movement
24	immune	immune_cell chemotaxis	123	eye	eye_Abnormality of eye movement
25	immune	immune_regulation of immune system process	124	neuron	neuron_neuron projection
26	immune	immune_Immune System	125	neuron	neuron_neuron part
27	immune	immune_G protein-coupled chemoattractant receptor activity	126	neuron	neuron_Demyelinating peripheral neuropathy
28	immune	immune_chemokine receptor activity	127	neuron	neuron_Polyneuropathy
29	immune	immune_C-C chemokine receptor activity	128	neuron	neuron_neurogenesis
30	immune	immune_chemokine binding	129	neuron	neuron_neuron development
31	immune	immune_C-C chemokine binding	130	neuron	neuron_neuron projection development
32	immune	immune_granulocyte chemotaxis	131	neuron	neuron_neuron differentiation
33	immune	immune_Abnormality of immune system physiology	132	neuron	neuron_generation of neurons
34	immune	immune_negative regulation of immune system process	133	neuron	neuron_Neurotransmitter release cycle
35	immune	immune_leukocyte differentiation	134	neuron	neuron_Transmission across Chemical Synapses
36	immune	immune_T cell activation	135	neuron	neuron_Neuronal System
37	immune	immune_T cell differentiation	136	neuron	neuron_synapse part
38	immune	immune_immune response	137	neuron	neuron_synapse
39	immune	immune_Abnormal immune system morphology	138	neuron	neuron_presynapse
40	immune	immune_Abnormal cellular immune system morphology	139	neuron	neuron_synaptic vesicle membrane
41	immune	immune_Abnormal leukocyte morphology	140	neuron	neuron_synaptic vesicle
42	immune	immune_Erythrocytes take up carbon dioxide and release oxygen	141	neuron	neuron_Sensorimotor neuropathy
43	immune	immune_O2/CO2 exchange in erythrocytes	142	neuron	neuron_neuron projection morphogenesis
44	immune	immune_mast cell activation	143	neuron	neuron_cell morphogenesis involved in neuron differentiation
45	immune	immune_Hematological neoplasm	144	neuron	neuron_neurotransmitter receptor complex
46	immune	immune_Abnormal erythrocyte morphology	145	neuron	neuron_synapse organization
47	immune	immune_embryonic hemopoiesis	146	neuron	neuron_postsynaptic specialization
48	immune	immune_myeloid cell development	147	neuron	neuron_neuron to neuron synapse
49	immune	immune_erythrocyte development	148	neuron	neuron_asymmetric synapse
50	immune	immune_Extramedullary hematopoiesis	149	neuron	neuron_Protein-protein interactions at synapses
51	immune	immune_Abnormality of T cell physiology	150	neuron	neuron_postsynaptic density
52	immune	immune_Hemolytic anemia	151	neuron	neuron_regulation of neurogenesis
53	immune	immune_heme biosynthetic process	152	neuron	neuron_postsynapse
54	immune	immune_heme metabolic process	153	neuron	neuron_postsynaptic density membrane
55	immune	immune_lymphocyte activation	154	neuron	neuron_postsynaptic specialization membrane
56	immune	immune_Autoimmune antibody positivity	155	neuron	neuron_regulation of trans-synaptic signaling
57	immune	immune_Cytokine Signaling in Immune system	156	neuron	neuron_modulation of chemical synaptic transmission
58	immune	immune_Subcutaneous hemorrhage	157	neuron	neuron_Dopamine Neurotransmitter Release Cycle
59	immune	immune_hematopoietic progenitor cell differentiation	158	neuron	neuron_neuronal cell body
60	immune	immune_hemoglobin complex	159	neuron	neuron_positive regulation of synaptic transmission
61	immune	immune_haptoglobin-hemoglobin complex	160	neuron	neuron_Glutamate Neurotransmitter Release Cycle
62	immune	immune_regulation of leukocyte migration	161	neuron	neuron_Upper motor neuron dysfunction
63	immune	immune_Abnormal macrophage morphology	162	neuron	neuron_postsynaptic membrane
64	immune	immune_positive regulation of immune system process	163	neuron	neuron_synaptic membrane
65	immune	immune_chemotaxis	164	neuron	neuron_synaptic signaling
66	immune	immune_regulation of leukocyte differentiation	165	neuron	neuron_Serotonin Neurotransmitter Release Cycle
67	immune	immune_Abnormal myeloid leukocyte morphology	166	neuron	neuron_Norepinephrine Neurotransmitter Release Cycle
68	immune	immune_regulation of hemopoiesis	167	neuron	neuron_Acetylcholine Neurotransmitter Release Cycle
69	immune	immune_Elevated erythrocyte sedimentation rate	168	neuron	neuron_protein localization to postsynaptic membrane
70	immune	immune_Abnormal erythrocyte sedimentation rate	169	neuron	neuron_neurotransmitter receptor transport, postsynaptic endosome to lysosome
71	immune	immune_lymphocyte differentiation	170	neuron	neuron_postsynaptic neurotransmitter receptor diffusion trapping
72	immune	immune_negative regulation of innate immune response	171	neuron	neuron_neurotransmitter receptor diffusion trapping
73	immune	immune_Adaptive Immune System	172	neuron	neuron_protein localization to postsynaptic specialization membrane
74	immune	immune_leukocyte activation	173	neuron	neuron_neurotransmitter receptor localization to postsynaptic specialization membrane
75	immune	immune_neutrophil chemotaxis	174	neuron	neuron_regulation of postsynaptic membrane neurotransmitter receptor levels
76	immune	immune_macrophage migration	175	neuron	neuron_receptor localization to synapse
77	eye	eye_Optic neuropathy	176	neuron	neuron_neuron projection guidance
78	eye	eye_Slow decrease in visual acuity	177	neuron	neuron_Neurodevelopmental abnormality
79	eye	eye_Retinal telangiectasia	178	neuron	neuron_regulation of neuronal synaptic plasticity
80	eye	eye_Leber optic atrophy	179	neuron	neuron_Optic neuropathy
81	eye	eye_Central retinal vessel vascular tortuosity	180	neuron	neuron_Sensory neuropathy
82	eye	eye_Retinal arterial tortuosity	181	neuron	neuron_Peripheral neuropathy
83	eye	eye_Retinal vascular tortuosity	182	neuron	neuron_synaptic transmission, glutamatergic
84	eye	eye_Progressive visual loss	183	neuron	neuron_Abnormality of neuronal migration
85	eye	eye_Abnormal retinal artery morphology	184	neuron	neuron_neuron projection organization
86	eye	eye_Abnormal visual electrophysiology	185	neuron	neuron_Na+/Cl- dependent neurotransmitter transporters
87	eye	eye_Retinal dystrophy	186	neuron	neuron_Neurodevelopmental delay
88	eye	eye_Phototransduction	187	neuron	neuron_regulation of neurotransmitter levels
89	eye	eye_Inactivation, recovery and regulation of the phototransduction cascade	188	neuron	neuron_Neurological speech impairment
90	eye	eye_The phototransduction cascade	189	neuron	neuron_vesicle-mediated transport in synapse
91	eye	eye_Visual field defect	190	neuron	neuron_synaptic vesicle cycle
92	eye	eye_Abnormality of visual evoked potentials	191	neuron	neuron_regulation of synaptic vesicle cycle
93	eye	eye_Visual loss	192	neuron	neuron_trans-synaptic signaling
94	eye	eye_Abnormal retinal vascular morphology	193	neuron	neuron_chemical synaptic transmission
95	eye	eye_Abnormality of retinal pigmentation	194	neuron	neuron_anterograde trans-synaptic signaling
96	eye	eye_Abnormality of the vasculature of the eye	195	neuron	neuron_signal release from synapse
97	eye	eye_Abnormal retinal morphology	196	neuron	neuron_neurotransmitter secretion
98	eye	eye_Constriction of peripheral visual field	197	neuron	neuron_neurotransmitter transport
99	eye	eye_visual perception	198	neuron	neuron_synaptic vesicle exocytosis

Supplementary Table 10: Full list of GO terms and corresponding p-values appearing in invariant clusters but not in any individual clusters for scGESTALT dataset. The significant GO terms were obtained from g:Profiler [17], where hypergeometric distribution is adopted for performing statistical test and the p-values are adjusted based on g:SCS algorithm [18]

**GO term**	**cluster,p-value**
neuron projection	(c10,1.16e-02),(c23,1.05e-02),(c20,2.60e-03)
Developmental regression	(c33,2.09e-02),(c30,3.59e-02)
neuron projection development	(c33,8.45e-04)
Acetylcholine Neurotransmitter Release Cycle	(c17,1.39e-02)
erythrocyte homeostasis	(c22,2.02e-05)
Neurotransmitter release cycle	(c23,1.34e-02)
presynaptic active zone	(c33,3.88e-02)
neuron development	(c33,5.65e-04),(c4,6.96e-03)
regulation of neurotransmitter levels	(c4,3.83e-02)
animal organ development	(c32,6.68e-04)
heme-copper terminal oxidase activity	(c10,2.60e-02)
Global developmental delay	(c10,9.72e-03)
embryo development	(c22,6.09e-05)
Abnormal erythrocyte morphology	(c3,8.46e-03)
Polyneuropathy	(c33,9.37e-03)
synaptic vesicle cycle	(c30,1.94e-03)
trans-synaptic signaling	(c30,1.58e-03)
peripheral nervous system neuron development	(c16,2.28e-02)
synapse part	(c20,2.66e-02)
neuron projection cytoplasm	(c10,3.82e-03)
erythrocyte development	(c19,1.49e-02),(c22,4.11e-02),(c32,1.28e-02)
developmental process	(c31,2.49e-04),(c4,1.85e-03)
Serotonin Neurotransmitter Release Cycle	(c17,1.11e-02)
neuron projection guidance	(c4,1.11e-02)
Neurodevelopmental delay	(c10,2.95e-02)
myeloid cell homeostasis	(c3,5.89e-03),(c22,4.34e-05)
myeloid cell development	(c8,3.48e-02),(c32,5.34e-03)
synaptic signaling	(c30,1.75e-03)
Abnormal cellular immune system morphology	(c3,4.18e-02),(c32,1.65e-04)
peripheral nervous system neuron differentiation	(c16,2.28e-02)
Transmission across Chemical Synapses	(c23,4.75e-04)
neurotransmitter transport	(c4,3.61e-02)
synaptic vesicle	(c23,5.08e-04)
regulation of developmental process	(c16,1.72e-02),(c22,4.64e-03)
oxidoreductase activity, acting on a heme group of donors	(c10,2.60e-02)
anterograde trans-synaptic signaling	(c30,1.50e-03)
Optic neuropathy	(c33,8.66e-04)
chemical synaptic transmission	(c30,1.50e-03)
positive regulation of developmental process	(c16,4.12e-02)
Norepinephrine Neurotransmitter Release Cycle	(c17,1.11e-02)
neuronal cell body	(c9,1.14e-02)
myeloid cell differentiation	(c22,1.45e-03)
Abnormal immune system morphology	(c19,2.10e-03),(c32,2.33e-04)
generation of neurons	(c33,3.62e-03)
system development	(c31,1.03e-03),(c8,1.37e-02),(c32,3.78e-02)
signal release from synapse	(c4,1.85e-02)
Hematological neoplasm	(c3,2.01e-03)
neurogenesis	(c33,7.82e-03)
Neurotransmitter uptake and metabolism In glial cells	(c2,1.77e-02)
vesicle-mediated transport in synapse	(c30,1.94e-03)
synapse	(c20,1.49e-02)
embryo development ending in birth or egg hatching	(c31,1.60e-03),(c22,3.11e-04)
presynapse	(c33,4.28e-03),(c23,8.44e-04)
anatomical structure development	(c31,1.32e-04)
Neuronal System	(c23,1.85e-02)
Delayed speech and language development	(c10,3.85e-03)
erythrocyte differentiation	(c22,1.88e-05)
chordate embryonic development	(c31,1.52e-03),(c22,2.98e-04)
neurotransmitter secretion	(c4,1.85e-02)
Neurological speech impairment	(c33,2.01e-02)
oxidoreductase activity, acting on a heme group of donors, oxygen as acceptor	(c10,2.60e-02)
synaptic vesicle exocytosis	(c30,6.84e-03)
definitive hemopoiesis	(c19,3.68e-02)
neuron part	(c20,9.57e-04)
immune system process	(c8,4.81e-03),(c32,9.54e-04)
regulation of cell differentiation	(c16,6.97e-03)

Supplementary Table 11: Full list of GO terms appearing in invariant clusters but not in any individual clusters for ScarTrace dataset. The significant GO terms were obtained from g:Profiler [17], where hypergeometric distribution is adopted for performing statistical test and the p-values are adjusted based on g:SCS algorithm [18]
.

GO term	cluster,p-value	GO term	cluster,p-value
granulocyte chemotaxis	(c9,9.00e-03)	Hematological neoplasm	(c15,1.22e-02)
Abnormality of the optic disc	(c47,1.02e-04)	postsynaptic membrane	(c7,7.61e-04)
presynapse	(c7,6.46e-05),(c4,2.74e-02),(c22,2.99e-03),(c47,4.78e-03)	neuron development	(c3,1.15e-02)
Neurotransmitter release cycle	(c30,1.19e-03),(c20,1.80e-02)	synaptic vesicle exocytosis	(c21,8.46e-04)
embryonic hemopoiesis	(c44,1.72e-02),(c37,1.03e-02),(c46,4.71e-02)	Subcutaneous hemorrhage	(c29,2.85e-02)
regulation of postsynaptic membrane neurotransmitter receptor levels	(c2,4.26e-02)	Activation of the phototransduction cascade	(c47,1.90e-03)
Abnormal lymphocyte physiology	(c43,8.95e-03)	Abnormal lymphocyte count	(c19,2.73e-02)
Abnormal retinal morphology	(c29,4.22e-02)	Acetylcholine Neurotransmitter Release Cycle	(c21,2.94e-02)
neuron part	(c12,5.05e-03),(c16,4.98e-03)	Sensorimotor neuropathy	(c52,2.34e-02)
response to chemokine	(c19,2.40e-02),(c9,2.88e-03),(c43,2.19e-02)	Optic disc hypoplasia	(c47,1.40e-03)
G protein-coupled chemoattractant receptor activity	(c9,3.05e-03),(c43,4.26e-02)	synapse part	(c12,1.18e-02)
erythrocyte development	(c33,2.67e-02),(c44,2.66e-02)	postsynaptic specialization	(c7,4.86e-02)
Abnormality of retinal pigmentation	(c47,1.19e-02)	cell chemotaxis	(c19,1.23e-03)
lymphocyte activation	(c19,1.48e-02)	Glutamate Neurotransmitter Release Cycle	(c7,5.28e-03)
neurogenesis	(c18,1.60e-02),(c28,3.24e-02)	synaptic signaling	(c7,3.99e-02)
Neurodevelopmental delay	(c12,3.92e-02),(c21,2.47e-02)	Innate Immune System	(c43,4.06e-02)
protein localization to postsynaptic specialization membrane	(c2,1.55e-02)	receptor localization to synapse	(c2,4.98e-02)
positive regulation of synaptic transmission	(c7,4.36e-03)	Autoimmune antibody positivity	(c19,3.98e-02)
synaptic vesicle	(c30,1.76e-04),(c7,5.00e-04),(c22,3.05e-02)	photoreceptor cell outer segment organization	(c31,2.79e-04)
neuron differentiation	(c18,1.22e-02),(c2,2.91e-02)	Protein-protein interactions at synapses	(c7,4.05e-02)
regulation of leukocyte differentiation	(c8,4.79e-02),(c49,4.10e-02)	regulation of immune system process	(c19,4.24e-05)
chemokine binding	(c9,1.67e-03),(c43,2.61e-02)	Abnormality of immune system physiology	(c49,9.30e-04)
neuron projection guidance	(c1,2.33e-02)	neuronal cell body	(c7,8.91e-03)
T cell differentiation	(c19,4.34e-03)	synaptic membrane	(c7,7.33e-04)
positive regulation of immune system process	(c49,2.15e-02)	Visual impairment	(c31,1.52e-02)
C-C chemokine receptor activity	(c9,1.95e-03),(c43,2.96e-02)	generation of neurons	(c18,8.21e-03)
neurotransmitter receptor localization to postsynaptic specialization membrane	(c2,1.55e-02)	visual perception	(c31,3.32e-02)
cell morphogenesis involved in neuron differentiation	(c1,3.50e-02)	Neurological speech impairment	(c21,5.69e-06)
hemopoiesis	(c19,5.14e-03),(c37,2.11e-02),(c15,4.10e-04)	Abnormality of the vasculature of the eye	(c29,3.09e-02)
immune system development	(c19,8.14e-03),(c37,3.18e-02),(c15,6.79e-04)	asymmetric synapse	(c7,4.86e-02)
myeloid leukocyte migration	(c19,2.38e-03),(c9,3.28e-05),(c43,2.74e-04)	Demyelinating peripheral neuropathy	(c11,3.08e-02)
chemokine-mediated signaling pathway	(c19,2.09e-02),(c9,2.46e-03),(c43,1.90e-02)	neutrophil chemotaxis	(c9,6.51e-03)
Visual field defect	(c52,1.62e-02)	regulation of neurogenesis	(c7,4.30e-02)
synaptic vesicle membrane	(c30,3.29e-03),(c7,6.71e-03),(c22,9.14e-03)	neuron to neuron synapse	(c7,4.86e-02)
C-C chemokine binding	(c9,1.67e-03),(c43,2.61e-02)	leukocyte differentiation	(c19,9.16e-03)
leukocyte chemotaxis	(c19,5.48e-03),(c43,6.19e-04)	Abnormal erythrocyte sedimentation rate	(c43,1.65e-02)
neuron projection development	(c3,3.22e-02),(c1,6.08e-06)	neuron projection morphogenesis	(c1,6.23e-03)
regulation of hemopoiesis	(c8,1.10e-02),(c49,8.16e-03)	Optic atrophy	(c47,2.81e-03)
neuron projection	(c30,3.14e-04),(c5,2.79e-03),(c4,3.94e-03),(c16,3.02e-02)	Abnormal erythrocyte morphology	(c15,7.05e-03)
negative regulation of immune system process	(c19,2.98e-02),(c49,2.32e-03)	Erythrocytes take up carbon dioxide and release oxygen	(c46,2.27e-02)
neurotransmitter receptor diffusion trapping	(c2,4.68e-03)	regulation of synaptic vesicle cycle	(c21,4.98e-02)
Abnormal conjugate eye movement	(c13,6.12e-04),(c21,3.33e-02)	Serotonin Neurotransmitter Release Cycle	(c21,2.19e-02)
regulation of neurotransmitter levels	(c5,1.38e-02)	T cell activation	(c19,4.29e-03)
Abnormal leukocyte morphology	(c9,7.62e-03),(c15,1.56e-02)	leukocyte activation	(c9,8.84e-03)
Abnormality of the immune system	(c19,2.22e-02),(c49,3.65e-03),(c43,3.32e-02)	Abnormality of T cell physiology	(c44,3.91e-03)
leukocyte migration	(c19,1.75e-03),(c43,1.52e-03)	synapse organization	(c7,4.83e-02)
postsynaptic neurotransmitter receptor diffusion trapping	(c2,4.68e-03)	Progressive visual loss	(c11,3.96e-02)
macrophage chemotaxis	(c19,2.94e-02),(c43,2.87e-02)	Abnormality of the optic nerve	(c47,3.62e-04)
hematopoietic or lymphoid organ development	(c19,6.58e-03),(c37,2.63e-02),(c15,5.38e-04)	Abnormal macrophage morphology	(c49,4.62e-03)
Neuronal System	(c30,2.28e-02),(c21,5.00e-05),(c20,2.19e-03)	Polyneuropathy	(c11,4.25e-02)
chemokine receptor activity	(c9,3.05e-03),(c43,4.26e-02)	myeloid cell differentiation	(c37,5.16e-04)
myeloid cell development	(c33,2.53e-02),(c44,2.53e-02)	O2/CO2 exchange in erythrocytes	(c46,2.27e-02)
Abnormal cellular immune system morphology	(c9,7.62e-03),(c15,1.56e-02)	Extramedullary hematopoiesis	(c44,1.31e-02)
Abnormal immune system morphology	(c9,1.13e-02),(c15,1.92e-03)	Optic neuropathy	(c11,3.96e-03)
Transmission across Chemical Synapses	(c30,5.90e-04),(c20,9.52e-04)	postsynapse	(c7,2.80e-02)
Abnormal posterior eye segment morphology	(c47,5.63e-03)	Abnormal retinal vascular morphology	(c47,8.80e-03)
neurotransmitter receptor transport, postsynaptic endosome to lysosome	(c2,4.68e-03)	postsynaptic density	(c7,4.34e-02)
cellular response to chemokine	(c19,2.40e-02),(c9,2.88e-03),(c43,2.19e-02)	chemotaxis	(c49,1.45e-02)
Elevated erythrocyte sedimentation rate	(c43,1.65e-02)	immune system process	(c15,8.35e-04)
lymphocyte differentiation	(c19,7.01e-03),(c9,6.54e-03)	protein localization to postsynaptic membrane	(c2,3.69e-03)
Abnormal myeloid leukocyte morphology	(c8,1.98e-02),(c49,1.39e-03)	Abnormality of eye movement	(c21,4.37e-03)

References

[1] Wuming Gong, Il-Youp Kwak, Pruthvi Pota, Naoko Koyano-Nakagawa, and Daniel J Garry. DrImpute: imputing dropout events in single cell RNA sequencing data. *BMC bioinformatics*, 19(1):220, 2018.

[2] Mo Huang, Jingshu Wang, Eduardo Torre, Hannah Dueck, Sydney Shaffer, Roberto Bonasio, John I Murray, Arjun Raj, Mingyao Li, and Nancy R Zhang. Saver: gene expression recovery for single-cell rna sequencing. *Nature methods*, 15(7):539, 2018.

[3] Lihua Zhang and Shihua Zhang. Comparison of computational methods for imputing single-cell RNA-sequencing data. *IEEE/ACM transactions on computational biology and bioinformatics*, 2018.

[4] Kevin P Murphy. Conjugate Bayesian analysis of the Gaussian distribution. *def*, 1($2\sigma2$):16, 2007.

[5] Bushra Raj, Daniel E Wagner, Aaron McKenna, Shristi Pandey, Allon M Klein, Jay Shendure, James A Gagnon, and Alexander F Schier. Simultaneous single-cell profiling of lineages and cell types in the vertebrate brain. *Nature biotechnology*, 2018.

[6] Bushra Raj, James A Gagnon, and Alexander F Schier. Large-scale reconstruction of cell lineages using single-cell readout of transcriptomes and crispr–cas9 barcodes by scgestalt. *Nature protocols*, 13(11):2685, 2018.

[7] Irepan Salvador-Martínez, Marco Grillo, Michalis Averof, and Maximilian J Telford. Is it possible to reconstruct an accurate cell lineage using CRISPR recorders? *Elife*, 8:e40292, 2019.

[8] Edith M Ross and Florian Markowetz. OncoNEM: inferring tumor evolution from single-cell sequencing data. *Genome biology*, 17(1):69, 2016.

[9] Hamim Zafar, Nicholas Navin, Ken Chen, and Luay Nakhleh. SiCloneFit: Bayesian inference of population structure, genotype, and phylogeny of tumor clones from single-cell genome sequencing data. *Genome Research*, 2019.

[10] Nicolas Diotel, Rebecca Rodriguez Viales, Olivier Armant, Martin März, Marco Ferg, Sepand Rastegar, and Uwe Strähle. Comprehensive expression map of transcription regulators in the adult zebrafish telencephalon reveals distinct neurogenic niches. *Journal of Comparative*

Neurology, 523(8):1202–1221, 2015.

[11] Mario F Wullimann, Thomas Mueller, Martin Distel, Andreas Babaryka, Benedikt Grothe, and Reinhard W Köster. The long adventurous journey of rhombic lip cells in jawed vertebrates: a comparative developmental analysis. *Frontiers in neuroanatomy*, 5:27, 2011.

[12] Miki Takeuchi, Shingo Yamaguchi, Yoshimasa Sakakibara, Takuto Hayashi, Koji Matsuda, Yuichiro Hara, Chiharu Tanegashima, Takashi Shimizu, Shigehiro Kuraku, and Masahiko Hibi. Gene expression profiling of granule cells and Purkinje cells in the zebrafish cerebellum. *Journal of Comparative Neurology*, 525(7):1558–1585, 2017.

[13] Thomas Mueller and Mario Wullimann. *Atlas of early zebrafish brain development: a tool for molecular neurogenetics.* Academic Press, 2015.

[14] Victor Muthu, Helen Eachus, Pam Ellis, Sarah Brown, and Marysia Placzek. Rx3 and Shh direct anisotropic growth and specification in the zebrafish tuberal/anterior hypothalamus. *Development*, 143(14):2651–2663, 2016.

[15] Olivier Armant, Martin März, Rebecca Schmidt, Marco Ferg, Nicolas Diotel, Raymond Ertzer, Jan Christian Bryne, Lixin Yang, Isabelle Baader, Markus Reischl, et al. Genome-wide, whole mount in situ analysis of transcriptional regulators in zebrafish embryos. *Developmental biology*, 380(2):351–362, 2013.

[16] Anna Alemany, Maria Florescu, Chloé S Baron, Josi Peterson-Maduro, and Alexander Van Oudenaarden. Whole-organism clone tracing using single-cell sequencing. *Nature*, 556(7699):108, 2018.

[17] Jüri Reimand, Tambet Arak, Priit Adler, Liis Kolberg, Sulev Reisberg, Hedi Peterson, and Jaak Vilo. g: Profiler—a web server for functional interpretation of gene lists (2016 update). *Nucleic acids research*, 44(W1):W83–W89, 2016.

[18] Jüri Reimand, Meelis Kull, Hedi Peterson, Jaanus Hansen, and Jaak Vilo. g: Profiler—a web-based toolset for functional profiling of gene lists from large-scale experiments. *Nucleic acids research*, 35(suppl_2):W193–W200, 2007.
