# Supplementary material for: Single-cell lineage tracing by integrating CRISPR-Cas9 mutations with transcriptomic data
Source: Nat Commun. 2020 Jun 16;11:3055. doi: 10.1038/s41467-020-16821-5 (PMC7298005; doi:10.1038/s41467-020-16821-5)
Supplement: Supplementary file 3 — Reporting Summary [file 41467_2020_16821_MOESM3_ESM.pdf]

nature research

Corresponding author(s): Ziv Bar-Joseph

Last updated by author(s): May 13, 2020

Reporting Summary

Nature Research wishes to improve the reproducibility of the work that we publish. This form provides structure for consistency and transparency in reporting. For further information on Nature Research policies, see Authors & Referees and the Editorial Policy Checklist.

Statistics

For all statistical analyses, confirm that the following items are present in the figure legend, table legend, main text, or Methods section.

n/a	Confirmed	
☒	☐	The exact sample size (*n*) for each experimental group/condition, given as a discrete number and unit of measurement
☒	☐	A statement on whether measurements were taken from distinct samples or whether the same sample was measured repeatedly
☐	☒	The statistical test(s) used AND whether they are one- or two-sided<br>*Only common tests should be described solely by name; describe more complex techniques in the Methods section.*
☐	☒	A description of all covariates tested
☐	☒	A description of any assumptions or corrections, such as tests of normality and adjustment for multiple comparisons
☐	☒	A full description of the statistical parameters including central tendency (e.g. means) or other basic estimates (e.g. regression coefficient) AND variation (e.g. standard deviation) or associated estimates of uncertainty (e.g. confidence intervals)
☐	☒	For null hypothesis testing, the test statistic (e.g. *F*, *t*, *r*) with confidence intervals, effect sizes, degrees of freedom and *P* value noted<br>*Give P values as exact values whenever suitable.*
☒	☐	For Bayesian analysis, information on the choice of priors and Markov chain Monte Carlo settings
☐	☒	For hierarchical and complex designs, identification of the appropriate level for tests and full reporting of outcomes
☒	☐	Estimates of effect sizes (e.g. Cohen's *d*, Pearson's *r*), indicating how they were calculated

Our web collection on statistics for biologists contains articles on many of the points above.

Software and code

Policy information about availability of computer code

Data collection	We did not use any software for data collection.
Data analysis	We wrote the LinTIMaT, visualization and GO analysis scripts. As part of LinTIMaT, we implemented Fitch's algorithm, BHC algorithm, etc. We used PhyloNet (v3.6.8) and Apache Commons Math (v3.6.1) package in LinTIMaT. LinTIMaT is freely available at https://github.com/jessica1338/LinTIMaT under the MIT license. Our visualization script is modified from the visualization scripts of scGESTALT.

For manuscripts utilizing custom algorithms or software that are central to the research but not yet described in published literature, software must be made available to editors/reviewers. We strongly encourage code deposition in a community repository (e.g. GitHub). See the Nature Research guidelines for submitting code & software for further information.

Data

Policy information about availability of data

All manuscripts must include a data availability statement. This statement should provide the following information, where applicable:

- Accession codes, unique identifiers, or web links for publicly available datasets
- A list of figures that have associated raw data
- A description of any restrictions on data availability

We did not collect any new data for the paper. All data used is referenced and available at the source. All the datasets used in this study have been previously deposited in the Gene Expression Omnibus under accession numbers GSE77944, GSE105010 and GSE102990. Lineage trees are available for exploring at https://jessica1338.github.io/LinTIMaT/

Field-specific reporting

Please select the one below that is the best fit for your research. If you are not sure, read the appropriate sections before making your selection.

☒ Life sciences ☐ Behavioural & social sciences ☐ Ecological, evolutionary & environmental sciences

For a reference copy of the document with all sections, see nature.com/documents/nr-reporting-summary-flat.pdf

Life sciences study design

All studies must disclose on these points even when the disclosure is negative.

Sample size	We used prior data. Study design is based on the decision made by the authors of these prior papers.
Data exclusions	For ScarTrace, we only used the fishes for which cells were sampled from brain and eyes. For scGESTALT we excluded Fish 2 when learning the invariant lineage tree because it had the lowest ARI based on the clustering performed in the original paper. Exclusions based on cell types and agreement were pre-determined.
Replication	We tested the method on multiple datasets from each study and using different platform data. Replication experiments confirmed the robustness of our method.
Randomization	N/A
Blinding	N/A

Reporting for specific materials, systems and methods

We require information from authors about some types of materials, experimental systems and methods used in many studies. Here, indicate whether each material, system or method listed is relevant to your study. If you are not sure if a list item applies to your research, read the appropriate section before selecting a response.

Materials & experimental systems

n/a	Involved in the study	
☒	☐	Antibodies
☒	☐	Eukaryotic cell lines
☒	☐	Palaeontology
☒	☐	Animals and other organisms
☒	☐	Human research participants
☒	☐	Clinical data

Methods

n/a	Involved in the study	
☒	☐	ChIP-seq
☒	☐	Flow cytometry
☒	☐	MRI-based neuroimaging
